# DNA Damage-Induced Inflammatory Microenvironment and Adult Stem Cell Response

**DOI:** 10.3389/fcell.2021.729136

**Published:** 2021-10-08

**Authors:** Davide Cinat, Robert P. Coppes, Lara Barazzuol

**Affiliations:** ^1^Department of Biomedical Sciences of Cells and Systems, University Medical Center Groningen, University of Groningen, Groningen, Netherlands; ^2^Department of Radiation Oncology, University Medical Center Groningen, University of Groningen, Groningen, Netherlands

**Keywords:** DNA damage, inflammation, microenvironment, immune response, stem cells, cancer

## Abstract

Adult stem cells ensure tissue homeostasis and regeneration after injury. Due to their longevity and functional requirements, throughout their life stem cells are subject to a significant amount of DNA damage. Genotoxic stress has recently been shown to trigger a cascade of cell- and non-cell autonomous inflammatory signaling pathways, leading to the release of pro-inflammatory factors and an increase in the amount of infiltrating immune cells. In this review, we discuss recent evidence of how DNA damage by affecting the microenvironment of stem cells present in adult tissues and neoplasms can affect their maintenance and long-term function. We first focus on the importance of self-DNA sensing in immunity activation, inflammation and secretion of pro-inflammatory factors mediated by activation of the cGAS-STING pathway, the ZBP1 pathogen sensor, the AIM2 and NLRP3 inflammasomes. Alongside cytosolic DNA, the emerging roles of cytosolic double-stranded RNA and mitochondrial DNA are discussed. The DNA damage response can also initiate mechanisms to limit division of damaged stem/progenitor cells by inducing a permanent state of cell cycle arrest, known as senescence. Persistent DNA damage triggers senescent cells to secrete senescence-associated secretory phenotype (SASP) factors, which can act as strong immune modulators. Altogether these DNA damage-mediated immunomodulatory responses have been shown to affect the homeostasis of tissue-specific stem cells leading to degenerative conditions. Conversely, the release of specific cytokines can also positively impact tissue-specific stem cell plasticity and regeneration in addition to enhancing the activity of cancer stem cells thereby driving tumor progression. Further mechanistic understanding of the DNA damage-induced immunomodulatory response on the stem cell microenvironment might shed light on age-related diseases and cancer, and potentially inform novel treatment strategies.

## Introduction

Stem cells are undifferentiated cells essential for tissue growth and maintenance (Blanpain and Simons, 2013). They can be classified according to their origin in embryonic stem cells, induced pluripotent stem cells (iPSCs) and tissue-specific stem cells (also known as adult or somatic stem cells) (Shevde, 2012). Embryonic and iPSCs are pluripotent stem cells, derived from an early stage embryo and reprogramming of somatic cells, respectively, able to differentiate into any specialized tissue cell type (Kingham and Oreffo, 2013). On the other hand, tissue-specific stem cells are multi- or unipotent cells able to give rise to specialized cell type(s) present in a specific tissue and belonging to a particular lineage. Tissue-specific stem cells are present in small numbers in several adult tissues or organs ensuring tissue homeostasis and regeneration upon damage (Wagers and Weissman, 2004). Similarly to normal tissues, most tumors also possess a population of cells characterized by stem cell-like features that are defined as cancer stem cells (CSCs) (Coppes and Dubrovska, 2017). Like normal stem cells, CSCs have the ability to self-renew and generate differentiated cell types, which foster the growth and maintenance of many types of neoplasms alongside being often attributed to treatment resistance and cancer recurrence (Vitale et al., 2017).

**FIGURE 1:**
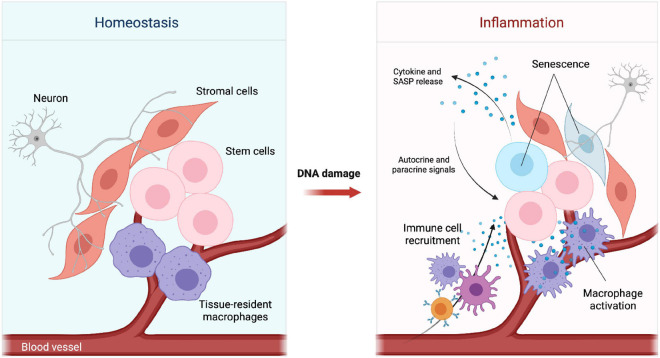
Changes in the stem cell microenvironment upon DNA damage. Simplified schematic representation of the tissue microenvironment during homeostasis **(Left)** and inflammation **(Right)**. DNA damage can impair tissue homeostasis by promoting senescence, cytokine and SASP release. Cytokines and chemokines present in the microenvironment can lead to recruitment of immune cells and activation of tissue-resident macrophages.

Stem cells constantly receive signals from the surrounding microenvironment, also known as the stem cell niche, a ‘home’ that supports the maintenance and proper function of stem cells to ensure tissue homeostasis and respond to damage (Jones and Wagers, 2008). These niche signals can be either extrinsic, mediated by secreted factors, cell surface molecules/receptors, cell-cell interactions and gap junctions, or intrinsic resulting in persistent intracellular changes in the stem cell epigenetic profile and metabolism (Pennings et al., 2018). Additionally, the stem cell microenvironment is composed by specific cells, often distributed in a defined spatial order, such as differentiated cells, stromal cells, immune cells, vasculature- and nervous system-related cells (Figure 1, left panel). These cells work together to ensure structure and appropriate reception of local as well as systemic signals (Lane et al., 2014).

The non-cellular physical properties of the microenvironment itself, such as the extra-cellular matrix (ECM) molecule composition and oxygen levels, can also affect stem cell behavior by affecting stem cell related pathways. A classic example of this is the role of the YAP/TAZ signaling pathway in mechanotransduction (Dupont et al., 2011). While hypoxia inducible factors (HIFs) have been shown to modulate other stem cell-related pathways, such as Notch signaling (Keith and Simon, 2007) and autophagy (Li et al., 2015).

The structure and function of the stem cell microenvironment have been extensively reviewed in Jones and Wagers (2008). Additionally, notions described for the hematopoietic stem cell (HSC) niche (Mendelson and Frenette, 2014; Crane et al., 2017; Pinho and Frenette, 2019), such as the maintenance of HSCs via specific factors secreted by endothelial and stromal cells as well as immune cells and sympathetic nerve fibers, can be applied to solid tissues. Importantly, the stem cell microenvironment has been shown to be a dynamic compartment rapidly adapting in response to insults, diseases (including oncogenesis) and aging (Scadden, 2006). Aging is a pleiotropic process characterized by multiple factors, including increased levels of DNA damage (Schumacher et al., 2021) due to various sources (as described in the following section) coupled with a reduced cellular DNA repair capacity.

Throughout life stem cells are significantly exposed to DNA damage due to their longevity and functional requirements, such as ensuring tissue homeostasis and replenishment of damaged or lost cells via prolonged proliferation (self-renewal) and differentiation (Mandal et al., 2011; Schumacher et al., 2021). In particular, cell proliferation is intrinsically related to replication stress, a phenomenon characterized by DNA synthesis slow down and stalled replication forks that in turn can result in DNA damage as previously shown in aged HSCs (Flach et al., 2014).

In this review, we describe how the recently discovered immunomodulatory responses initiated by DNA damage can alter different aspects of the stem cell microenvironment thereby affecting the function of both tissue-specific and cancer stem cells. The main cytosolic nucleic acid sensing pathways activated upon cytosolic DNA and RNA recognition and involved mechanisms, which can induce microenvironmental changes that affect stem cell function, are discussed. Additionally, an overview of immune cell infiltration and the importance of DNA damage-induced cellular senescence in tissue homeostasis, stem cell regeneration potential and CSC recognition is provided.

## DNA Damage in Adult Stem Cells

Different endogenous sources of DNA damage can affect adult stem cells and their microenvironment, such as reactive oxygen species (ROS) produced by metabolic intermediates and dysfunctional mitochondria, alcohol and endogenous aldehydes, glycolytic by-products and advanced glycation end products, replication stress depending on the proliferation status of the cells, transcriptional disruption and telomere shortening. Importantly, although it is difficult to reliably assess such endogenous sources of DNA damage, they are thought to increase with age (Chaudhuri et al., 2018; Schumacher et al., 2021). As a result of such physiological cellular processes, in a day each cell may be exposed to nearly 100,000 DNA lesions, including different types of base modifications, single-strand breaks (SSBs) and double-strand breaks (DSBs) (Madabhushi et al., 2014). Additionally, external sources can lead to DNA damage, such as ionizing radiation (most commonly UV and X-rays) and certain chemicals. This is especially relevant for CSCs and normal tissue stem cells co-exposed to DNA damaging cancer therapies, such as radiotherapy and many chemotherapeutic agents.

In response to DNA damage cells initiate a coordinated series of events known as the DNA damage response (DDR), which encompasses various DNA repair pathways, cell cycle checkpoints and cell death pathways, and extensively described in Jackson and Bartek (2009). The DDR initiates with the sensing of DNA damage by protein complexes and kinases, and the subsequent signaling mediated by post-translational modifications, such as protein phosphorylation. Although these complex molecular mechanisms can vary between different types of somatic cells and stem cells (Vitale et al., 2017), the main DNA repair pathways are usually conserved and depending on the type of DNA lesion comprise: base excision repair (BER) and single strand break repair (SSBR), which promote the repair of small DNA lesions, such as base modifications and SSBs, through the excision of damaged bases; nucleotide excision repair (NER), which promotes the repair of DNA lesions such as adducts and structures that distort the DNA double helix; DNA mismatch repair (MMR), essential for the correction of base mismatches and small insertions or deletions; non-homologous end joining (NHEJ) and homologous recombination (HR), which are the classical pathways involved in DSB repair (Jackson and Bartek, 2009; Scully et al., 2019). Collectively with DNA repair, DNA damage signaling can lead to the activation of cell cycle checkpoints, which are points throughout the cell cycle in which movement is paused or slowed down to allow time for the cell to repair the damage, or to the induction of cell death, mainly by apoptosis and necrosis, or to an irreversible state of growth arrest so-called cellular senescence (Jackson and Bartek, 2009).

As stem cells age, DNA damage coupled with a reduced DNA repair capacity has been shown to contribute to the development of age-related disorders from cancer to tissue degeneration (Behrens et al., 2014). Oncogenesis is often a result of aging due to DNA damage misrepair and the consequent accumulation of mutations (Jeggo et al., 2016). A decline in stem cell function has also been related to defects in different DDR components, which in the HSC system cause reduced self-renewal and long-term exhaustion leading to bone marrow failure and anemia (Vitale et al., 2017) or pre-mature differentiation in non-HSCs such as neural stem cells (NSCs) (Barazzuol et al., 2017) and melanocyte stem cells (McSCs) (Inomata et al., 2009). Additionally, as a consequence of DNA damage, the microenvironment, the resulting signaling pathways and infiltrating cells adapt to this new state (Figure 1, right panel).

Although long-term self-renewal and differentiation capabilities are the defining features of stem cells, their regeneration potential is restricted to a definite number of times during the lifespan of an organism (Pazhanisamy, 2009). This can represent an important limitation; indeed, stem cells can be induced to self-renew more often upon damage or genotoxic stress, such as irradiation (Sémont et al., 2006). A prolonged activation of this process can inevitably lead to stem cell exhaustion and loss of tissue homeostasis maintenance (Pazhanisamy, 2009). Furthermore, many types of stem cells, including HSCs and NSCs (Barazzuol et al., 2017; Schumacher et al., 2021), reside in a quiescent state, which, although limits the amount of endogenous DNA damage (such as that caused by replication stress or metabolic by-products), may lead to the accumulation of DNA lesions due to the restricted availability of error prone NHEJ in non-cycling cells with subsequent stem cell functional impairment and premature aging (Schumacher et al., 2021). Moreover, DNA damage upon genotoxic stress has been shown to promote premature differentiation of McSCs, hair follicle stem cells and NSCs, thus preventing their expansion (Inomata et al., 2009; Matsumura et al., 2016; Barazzuol et al., 2017). However, it remains unclear whether DNA damage-induced premature differentiation can be linked to the DNA damage-mediated inflammatory process. Defects in DNA repair as in rare genetic disorders can also promote the decline of tissue stem cell functions leading to age-related diseases, such as bone marrow failure, and tumor formation via the generation of CSCs (Biechonski et al., 2017; Vitale et al., 2017; Tiwari and Wilson, 2019). For example, similarly to other stem cells, NSCs have the ability to migrate from their niche in order to differentiate and promote the repair of damaged brain tissue; however, abnormalities in this process may lead to stem cell transformation and glioblastoma formation (Vescovi et al., 2006). These events can be extended also to other tissues and organs, such as skin, liver, muscle and gut, whose repair depends on the activity of specific adult stem cells (Kenyon and Gerson, 2007).

## Interplay Between DNA Damage and Inflammation in the Stem Cell Microenvironment

One of the main roles of the immune system is to mediate the recognition of dangerous and invasive elements through the expression of specific pattern recognition receptors (PRRs). These elements can be distinguished in pathogen-associated molecular patterns (PAMPs) and damage-associated molecular patterns (DAMPs) (Gong et al., 2019). PAMPs are exogenous components that are unique to invading microorganisms, such as specific membrane-associated lipids, lipopolysaccharides (LPS) and lipoglycans (Silva-Gomes et al., 2014). In contrast, DAMPs are endogenous molecules released by damaged or dying cells and are not related to a pathogen infection (Gong et al., 2019). Although PAMPs and DAMPs have different origins, the recognition of such molecules is mediated by similar PRRs, such as Toll-like receptors (TLRs), NOD-like receptors, intracellular nucleic acid-sensing receptors and C-type lectin receptors. Activation of these proteins is essential for the secretion of cytokines and attracting innate immune cells into the infected or damaged tissue (Gasteiger et al., 2017). The innate immune system can then intervene with cell-dependent mechanisms, such as phagocytosis, cytotoxicity and secreted factors, in order to eliminate the pathogens or damaged cells (Gasteiger et al., 2017). Several *in vitro* and *in vivo* studies have demonstrated that an aberrant activation of these mechanisms can trigger the host immune response leading to inflammatory events and autoimmune diseases (Nakad and Schumacher, 2016). Interestingly, receptors and adaptor proteins related to DAMP and PAMP recognition processes strongly overlap and interconnect often in a positive feedback loop. This tight relation might explain why infection as well as stress factors like DNA damage can trigger the activation of similar inflammatory pathways leading to pro-inflammatory cytokine release and immunity activation (Jounai et al., 2013; Foell et al., 2007; Paludan and Bowie, 2013).

It is well established that in normal physiological conditions DNA is largely located within the nucleus and mitochondria. However, DNA leakage within the cytosol can occur as result of adverse events, such as DNA damage, triggering the induction of specific cytosolic DNA sensors and activation of DAMP-related immune responses (Ishikawa et al., 2009; MacKenzie et al., 2017; Maekawa et al., 2019). Genotoxic events are usually accompanying by the formation of micronuclei into the cytoplasm, small extra-nuclear bodies formed by lagging chromosomes and chromosome fragments upon mitotic errors or DNA damage (Kwon et al., 2020). Importantly, due to defects in nuclear lamina organization (Hatch et al., 2013), the envelope of these isolated nuclear structures is fragile (Kwon et al., 2020). It has been shown that its rupture can lead to release of the micronuclear content into the cytoplasm with consequent chromothripsis (a mutational process characterized by the shattering and reassembly of a chromosome from a micronucleus) (Crasta et al., 2012; Koltsova et al., 2019). Alongside these processes, ruptured micronuclei have also been linked with the activation of various cytoplasmic nucleic acid sensors that boost the production of pro-inflammatory cytokines and activate the immune response (Crasta et al., 2012; MacKenzie et al., 2017). Interestingly, the activation of these pathways reflects a sign of microbial infections, which also alert the host innate immune system to mount a defense response.

These pro-inflammatory events can have important consequences on a variety of cell types, including stem cells, which function is modulated by their microenvironment (Voog and Jones, 2010). In fact, it has been shown that stem cell activity and their ability to self-renew can be strongly affected by either stress events, such as oxidative stress (Vono et al., 2018), or by chronic inflammatory diseases, such as bone marrow failure (Pronk et al., 2011; Vono et al., 2018). These events lead to the release of specific cytokines and chemokines into the microenvironment with consequent abnormal stem cell proliferation, mobilization and differentiation as well as premature quiescence and self-renewal decline (Jahandideh et al., 2020). Thus, excessive production and release of pro-inflammatory cytokines upon DNA damage can highly affect the stem cell regeneration capacity contributing to long-term dysfunction in aging tissues, including skin, bone marrow and adipose tissue (Crop et al., 2010; Shin et al., 2017; Hormaechea-Agulla et al., 2020).

Like normal tissue-specific stem cells, also CSCs can be influenced by changes in their surrounding microenvironment; indeed, it has been shown that pro-inflammatory stimuli are an essential component of the CSC niche (Zhang S. et al., 2018) able to potentially alter their function. The presence of inflammatory cytokines can have conflicting effects on CSCs. Growing evidence suggest that inflammation may be an important source of tumor progression and CSC expansion (Jeong et al., 2018), while other studies showed that specific type of cytokines, in particular interferons, can exert anti-tumor activity and obstruct angiogenesis (Martin-Hijano and Sainz, 2020).

### Cytosolic DNA Sensors and STING Activation

Cytokines are in fact powerful mediators of stem cell function and their wide range of effects highlights the importance of DNA-sensing pathways, and activation and release of inflammatory molecules. In particular, one of the main adaptor proteins activated upon cytosolic DNA recognition and essential for the initiation of these inflammatory responses is stimulator of interferon genes (STING) (Ishikawa and Barber, 2008).

**FIGURE 2:**
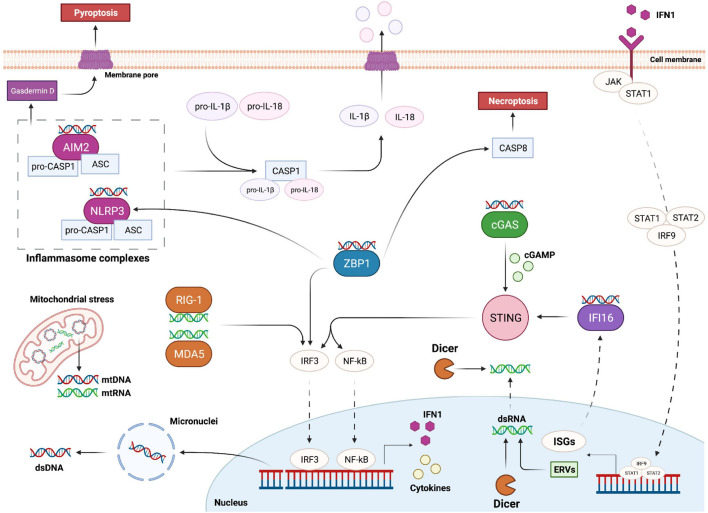
Overview of the main pathways responsible for cytosolic nucleic acid recognition. DNA damage can trigger the formation of micronuclei and the release of double stranded DNA (dsDNA) into the cytoplasm. Mitochondria can also be a source of cytoplasmic DNA and RNA upon genomic stress. AIM2 and NLRP3 are part of two distinct inflammasome complexes, responsible for the bioactivation of Caspase-1 (CASP1); activated CASP1 in turn cleaves and promotes the activation of IL-1β and IL-18, which leave the cell upon inflammasome-mediated pyroptosis. cGAS is the main protein responsible for cytoplasmic dsDNA recognition; upon dsDNA binding cGAS promotes the formation of cGAMP, which binds and activates STING; STING in turn promotes the activation of IRF3 and NF-kB transcription factors responsible for the expression of IFN1 and various cytokines. IFN1 is then able to leave the cell and interact with its receptor; this interaction leads to STAT1, STAT2 and IRF9 complex (ISGF3) translocation into the nucleus and transcription of interferon stimulated genes (ISGs). ISGF3 activation can also lead to expression of ancestor endogenous retroviruses (ERVs) in form of double stranded RNA (dsRNA). Cytoplasmic dsRNA is recognized by RIG-1 and MDA5, which trigger the activation of IRF3. On the other hand, Dicer can sense and cleave both cytoplasmic and nucleic dsRNA. IFI16 is an ISG able to recognize dsDNA and activate STING. ZBP1 promotes IRF3 and NLRP3 activation, and Caspase-8 (CASP8)-mediated necroptosis upon recognition of Z forms of dsDNA.

STING has been identified as an essential component for the initiation of innate immune signaling processes following activation of pathways related to cytosolic nucleic acid recognition (Ishikawa and Barber, 2008). The activation of this endoplasmic reticulum adaptor protein is essential for the triggering of transcription pathways and efficient production of type 1 interferon (IFN1) in several mammalian cell types (Ishikawa and Barber, 2008; Ishikawa et al., 2009). Specifically, STING activation promotes the nuclear translocation of the transcription factors NF-κB and IRF3 leading to cytokine production and innate immune gene transcription with a strong impact on cell fate and tissue homeostasis (Yum et al., 2021) (Figure 2). The specific mechanisms and pathways involved in NF-κB and IRF3 activation, cytokine release, IFN1 expression and immunity regulation have been extensively reviewed in Liu et al. (2017) and Jefferies (2019).

Upon conformational changes, STING translocates to the Golgi apparatus thereby inducing the activation of IRF3 and NF-κB, which function together in order to promote the transcription of cytokines like IFN1 (Ablasser and Chen, 2019; Li and Chen, 2018).

Interferons are a group of cytokines able to modulate the immune response and related inflammatory events. IFN1 shows autocrine, paracrine, and systemic functions and upon interaction with its receptor is able to induce the expression of more than 200 interferon stimulated genes (ISGs), which reinforce the expression of IFN1 leading to inflammation and innate immune signaling activation (Dunphy et al., 2018; Lee and Ashkar, 2018; Martin-Hijano and Sainz, 2020). Although the secretion of IFN1 can be beneficial for the resolution of viral infection events, the chronic exposure to this cytokine can influence stem cell proliferation and thereby induce functional defects. For example, chronic expression of IFN1 as a consequence of DNA damage has been shown to be a critical mechanism that connects DNA damage accumulation with premature aging and inhibition of intestinal stem cell function both *in vitro* and *in vivo* (Yu et al., 2015). Furthermore, IFN1 was shown to be implicated in proliferation and exhaustion of HSCs, and suppression of IFN signaling safeguards stem cell self-renewal and differentiation capacity providing the basis for potential improvements of bone marrow transplantation (Sato et al., 2009). Due to the large spectrum of ISGs produced upon IFN1 activation, the presence of this cytokine might be critical for the treatment of some cancer types leading to the regression of CSCs and an overall decrease in tumor viability (Doherty et al., 2017; Martin-Hijano and Sainz, 2020). In fact, IFNs have been shown to impede tumor expansion by inducing prolonged cell cycle arrest and angiogenesis downregulation (Shang et al., 2011). Furthermore, IFNs are fundamental regulators of the immune response against tumors as exemplified by the IFN1-mediated immunogenicity of tumor cells increasing the immune system recognition (Martin-Hijano and Sainz, 2020).

One of the main proteins related to genotoxicity and mediation of immune responses upon cytosolic double-stranded DNA (dsDNA) recognition is the DNA-sensing enzyme cyclic guanosine monophosphate-adenosine monophosphate synthase (cGAS) (Table 1). Activated cGAS promotes the conversion of ATP and GTP into cyclin GMP-AMP (cGAMP), which binds and activates STING (Xia et al., 2016).

**TABLE 1:** List of DNA and RNA sensors mentioned in this review, the relative downstream effect and examples of stem cell types shown to express these sensors.

Sensor	Ligand	Source	Downstream effect	Stem cell type	References
cGAS	dsDNA	Virus infection; cytosolic self-DNA; micronuclei	STING activation; NF-kB activation; IFN1 expression	MSCs; HSCs; stem cell-like CD8+ T cells; embryonic stem cells	Yang et al., 2015; Li et al., 2020; Sharma et al., 2020; Zheng et al., 2020
IFI16	dsDNA or ssDNA	Virus infection; cytosolic self-DNA; micronuclei	Modulation of STING activity; NF-kB activation; IFN1 expression	Hair follicle stem cells; HSCs	Piccaluga et al., 2015; Orvain et al., 2020
ZBP1 (DAI)	Z-dsDNA or RNA	Virus infection; cytosolic self-DNA; endogenous RNA	IFN1 expression; cell death; NLRP3 activation	MSCs; intestinal stem cells	Wang et al., 2020; Zhao et al., 2020
AIM2	dsDNA	Virus infection; cytosolic self-DNA	AIM2 inflammasome complex assembly; IL-18 and IL-1β activation; cell death	Intestinal stem cells; epithelial stem cells; MSCs	Man et al., 2015; Yang et al., 2015; Naik et al., 2017
NLRP3	dsDNA	Virus infection; cytosolic self-DNA	NLRP3 inflammasome complex assembly; IL-18 and IL-1β activation; cell death	HSCs; MSCs; CSCs	Huang et al., 2017; Adamiak et al., 2020; Ahn et al., 2020
RLRs	dsRNA	Virus infection; endogenous dsRNA; ERV expression	IFN1 and ISGs expression	NSCs; MSCs; HSCs	Yang et al., 2013; Lin et al., 2019; Clapes et al., 2021
Dicer	dsRNA or ncRNA	Virus infection; endogenous dsRNA; ERV expression; miRNA	dsRNA cleavage and IFN1 repression	Embryonic stem cells; NSCs; CSCs; hair follicle stem cells; intestinal stem cells	Kawase-Koga et al., 2010; Iliou et al., 2014; Park et al., 2017; Vishlaghi and Lisse, 2020; Gurung et al., 2021

In addition to cGAS, other key DNA sensors are known to be important modulators of STING-dependent IFN1 production (Table 1). An example is the IFNγ-inducible protein 16 (IFI16), which can recognize both cytosolic and nuclear dsDNA (Almine et al., 2017). Importantly, this protein is not only a DNA sensor but it is itself an ISG, and the activation of this positive feedback loop can further enhance the inflammatory response and immune activation triggered by DNA damage and cytosolic DNA recognition (Dunphy et al., 2018) (Figure 2). Hence, excessive accumulation of IFI16 may have important consequences on IFN1-related autoimmune diseases, such as systemic lupus erythematosus and Sjogren syndrome (Li et al., 2019). Interestingly, a recent study showed that upon etoposide-induced DNA damage, DNA damage response factors, such ATM and PARP1, are able to activate p53 and TRAF6, which assemble into a protein complex together with IFI16 outside the nucleus. This complex can then activate STING in a non-canonical way leading to a more pronounced activation of NF-κB compared to IRF3 (Dunphy et al., 2018). Importantly, a higher induction of NF-κB can result in the expression of various pro-inflammatory related-genes, adhesion molecules and cell cycle regulators, such as IL-6, TNFα, RANTES, CXCL10, MMPs and BCL-2 family proteins (Liu et al., 2017; Dunphy et al., 2018).

Both canonical and non-canonical activations of NF-κB are known to be involved in immune and inflammatory responses. Promotion of this family of transcription factors leads to expression of a broad range of molecules leading to inflammation as well as cell survival, proliferation, angiogenesis, cell adhesion and metastasis (Liu et al., 2017). It has been observed that genotoxic stress and NF-κB autocrine and paracrine signaling are able to influence mesenchymal and hematopoietic stem cell characteristics affecting their proliferation capacity and regeneration potential (Ping et al., 2019). Furthermore, chronic exposure to inflammatory molecules induced through activation of NF-κB has been associated with uncontrolled NSC proliferation with consequent risk of mutagenesis and hence cancer development (Widera et al., 2008). NF-κB is also known to be a powerful activator of immune cells through the secretion of several chemokines and cytokines (Hayden et al., 2006). Immune cell infiltration in normal tissue can compromise tissue homeostasis and physiological function. Indeed, a recent study showed that loss of sensory neurons and decreased olfactory function in chronic rhinosinusitis can be linked to a prolonged inflammatory state alongside immune cell infiltration (Chen M. et al., 2019). NF-κB deregulation can indeed lead to overexpression of specific cytokines and chemokines, such as CCL19 and CCL20, with consequent recruitment and proliferation of macrophages and T cells causing a loss in olfactory mucosa horizontal basal stem cell regeneration potential and tissue homeostasis *in vivo* (Chen M. et al., 2019).

Recent studies have also shown a strong relation between specific types of stem cells and cytosolic DNA sensors (Liao et al., 2020). For example, proteins of the cGAS-STING pathway might be highly expressed in hematopoietic stem and progenitor cells (HSPCs) allowing a quick response to stress events and thus becoming critical components of HSPC-driven hematopoiesis (Qian et al., 2016; Liao et al., 2020). Hence, dysregulation of this pathway may lead to myeloid malignancies and inflammation-related diseases such as cardiovascular and metabolic diseases (Liao et al., 2020). Furthermore, recent works showed that activation of STING enhances the formation of a stem cell-like memory phenotype in T cells with a potential beneficial effect for immunotherapy (Li et al., 2020). STING may also play a pivotal role in the differentiation of neuronal progenitor cells (NPCs) into neurons by sensing DNA damage during brain development (Zhang et al., 2020).

Z-DNA binding protein 1 (ZBP1), also known as DLM-1 and DAI (DNA-dependent activator of IFN-regulatory factors), is another potential cytoplasmic recognition receptor able to sense nucleic acids from endogenous and exogenous sources (Table 1). Its role as Z-DNA/Z-RNA sensor has been long questioned and further studies are required to elucidate its exact function in inflammation and cell death. However, it has been observed that its activation upon cytoplasmic DNA recognition is sufficient to induce the expression of IFN1 through the activation of IRF3 and IRF7, independently from STING, as well as to induce necroptosis and the NLRP3 inflammasome complex (Takaoka et al., 2007; Kuriakose and Kanneganti, 2018) (Figure 2). Importantly, an uncontrolled execution of necroptosis and the release of immunogenic molecules by dying cells may result in detrimental inflammatory responses further driving autoimmune and chronic diseases such as skin inflammation, pulmonary diseases, kidney fibrosis, cardiovascular diseases, and neurodegenerative disorders (Choi et al., 2019; Devos et al., 2020).

### Inflammasome Activation and Cytokine Release

Activation of the inflammasomes, formed by innate immune system receptors and sensors (Guo et al., 2015), is another key event for the regulation and induction of inflammation. Upon sensing of PAMP and DAMP molecules inflammasomes are assembled by self-oligomerization into a caspase-1-activating scaffold leading to proinflammatory IL-1 family cleavage and bioactivation (Guo et al., 2015). Inflammasomes are mostly expressed by immune cells and the activation of caspase-1 is not only linked to pro-inflammatory cytokine promotion, but it is also a defining feature of a peculiar type of cell death called pyroptosis. This type of immunogenic cell death is caused by the formation of pores into the cell membrane that are generated upon cleavage of gasdermin D (Liu et al., 2016). This leads to a rapid plasma-membrane rupture and consequent release of proinflammatory molecules into the extracellular environment (Bergsbaken et al., 2009) (Figure 2). IL-1β together with IL-18 are the main pro-inflammatory cytokines produced upon inflammasome activation and after being released they can activate a broad spectrum of immunological and inflammatory responses (Strowig et al., 2012; Dinarello, 2018).

Different types of inflammasomes have been identified and described in literature (Schroder and Tschopp, 2010) and among them NLRP3 and AIM2 inflammasomes are the ones that are mostly related to DNA damage and cytokine release (Inoue and Shinohara, 2013; Wei et al., 2019). NLRP3 is part of the NLR protein family (He et al., 2016) and can be activated upon recognition of viral components as well as cytosolic danger signals (Zhao and Zhao, 2020). In the activated form, NLRP3 inflammasome is a multi-protein complex, constituted by NLRP3, ASC and procaspase-1, that is able to bioactivate IL-1β and IL-18 upon caspase-1 activation (Sharma and de Alba, 2021) (Figure 2). In particular, it has been observed that DNA damage in skin cells does not cause apoptosis but activation of a fibroblast-specific NLRP3 inflammasome and IL-1β secretion, which lead to defects in stem cell specification and consequent epithelial and dermal hyperplasia (Seldin and Macara, 2020). Similar results have been observed in human keratinocytes upon exposure to UV light. UV-induced DNA damage mediates an increase in NLRP3 gene expression and inflammatory cytokine production, such as IL-1β, IL-6 and TNFα, indicating that DNA damage induces the activation of NLRP3 inflammasome potentially leading to cutaneous tissue disorders (Hasegawa et al., 2016). Moreover, IL-1β and TNFα have been shown to affect adipogenic and osteogenic potential of murine mesenchymal stem cells (MSCs) *in vitro*, which might correlate with collagen-induced arthritis *in vivo* (Sullivan et al., 2014).

Like NLRP3, AIM2 inflammasome activation is also important for the recognition of cytosolic dsDNA (Table 1). Indeed, AIM2 is an HIN-200 protein family member able to activate caspase-1 upon sensing of cytoplasmic DNA (Fernandes-Alnemri et al., 2009). Similarly to the NLRP3 inflammasome, interaction of AIM2 with ASC allows the activation of procaspase-1 leading to IL-1β and IL-18 activation and pyroptosis induction (Hornung et al., 2009; Sagulenko et al., 2013) (Figure 2). Interestingly, a recent study showed that AIM2 can also sense radiation-induced DNA damage into the nucleus of epithelial and bone marrow cells leading to AIM2 inflammasome assembly (Hu et al., 2016). As a consequence, AIM2 inflammasome can promote caspase-1 activation, immunogenic cell death and release of mature cytokines into the surrounding environment (Hu et al., 2016). As mentioned previously, CSCs also strongly depend on their microenvironment and events, like pyroptosis, with the consequent release of IL-1β and IL-18 into the extracellular environment might potentially lead to stimulation of dormant CSCs, leading to increased tumor treatment resistance and metastases (Tulotta et al., 2019; Van Gorp and Lamkanfi, 2019). This further supports pharmacologic inhibition of IL-1β as a potential cancer treatment strategy (Tulotta et al., 2019).

### Role of RIG-1-Like Receptors in dsRNA Sensing

The activation of nucleic acid sensors is not only limited to the recognition of cytosolic self-DNA but can also be induced by the presence of endogenous double-stranded RNA (dsRNA). Activation of retinoic acid-inducible gene 1 (RIG-1) like receptors (RLRs) can be triggered by both viral and host-derived RNAs leading to strong immune activation and inflammatory responses (Table 1) (Rehwinkel and Gack, 2020; Onomoto et al., 2021).

Several studies have shown a connection between the promotion of the IRF3-IFN1 axis and activation of RLRs in response to DNA damage. In particular, deregulation of both RIG-1 and MDA5 has been associated to autoimmune and inflammatory diseases induced by a STING-mediated IFN1 production (Ghosh et al., 2018; Onomoto et al., 2021).

Although why and how dsRNA is being released after DNA damage largely remains to be elucidated, recent studies showed an activation of latent endogenous retroviruses (ERVs) upon irradiation and DNA DSB formation. ERVs are originated from retroviruses that are thought to have infected early ancestor’s germ cells millions of years ago and most of these ERVs are normally silent or suppressed (Gao et al., 2021). However, it has been observed that after stress events, such as ionizing radiation-induced DNA damage, there is an activation of these dormant genes with consequent formation and release of dsRNA and IFN1 expression through the activation of STING (Lee et al., 2020). The promotion of these ERVs upon DNA damage may enhance the activation of transcription factors leading to innate immunity activation and secretion of molecules with similar consequences as the ones observed upon dsDNA recognition and cytosolic DNA sensor activation (Figure 2).

Another protein involved in dsRNA sensing is the endoribonuclease Dicer (Table 1), this enzyme is usually required for the processing and maturation of miRNAs (a class of small ncRNAs important for the regulation of gene expression at the post-transcriptional level) (Kuehbacher et al., 2007). However, recent studies showed its essential role also in the recognition and clearance of dsRNA localized in both the cytoplasm and the nucleus (Much et al., 2016; Burger et al., 2017). In fact, its downregulation has been shown to correlate with dsRNA accumulation and consequent IFN1 production *in vitro* (White et al., 2014). Interestingly, Dicer and its isoform (aviD) (Poirier et al., 2021) have been shown to be upregulated in NSCs, embryonic stem cells and adult intestinal stem cells (Kawase-Koga et al., 2010; Park et al., 2017; Gurung et al., 2021), and knock down of aviD has been shown to be related to higher levels of stem cell apoptosis upon viral infection (Poirier et al., 2021). Moreover, a DNA damage-inducible phospho-switch of Dicer has been linked with accumulation of this protein into the nucleus and consequent dsRNA clearance and prevention of RLR activation (Burger et al., 2017). Although central in dsRNA clearance upon viral infection, it remains unknown whether Dicer and aviD might play a role in the modulation of DNA damage-induced IFN1 expression in both normal and cancer stem cells.

### Role of Mitochondrial DNA in Inflammation and Immunity

Mitochondria are dynamic and essential organelles important for cellular bioenergetic maintenance, calcium metabolism and apoptotic processes (Detmer and Chan, 2007), and a proper function of this organelle is also important for stem cell self-renewal and differentiation (Zhang H. et al., 2018). Next to the DNA in the nucleus, also mitochondria contain several copies of their own circular DNA and changes in mitochondrial DNA (mtDNA) are often implicated in gene expression alterations, loss of tissue function, cancer and diseases (Singh et al., 2015; Castellani et al., 2020). DNA damage has been associated to mitochondrial dysfunction either via direct damage to the mtDNA or via depletion of nicotinamide adenine dinucleotide (NAD+) through activation of PARP1, a DDR protein that consumes NAD+, causing an imbalance in energy levels (Schumacher et al., 2021). Other processes such as DNA damage-associated defects in mitophagy and mtDNA replication might also contribute to mitochondrial dysfunction (Fang et al., 2014).

Mitochondrial disfunction and the consequent mtDNA leakage into the cytoplasm have been related to inflammatory responses upon activation of DNA sensor molecules. Similar to cytosolic self-DNA, recognition of cytosolic mtDNA can trigger the induction of inflammatory pathways, such as cGAS and NOD-like receptors leading to STING activation, promotion of IFNβ expression, release of NF-κB-related cytokines release as well as increased transcription and activation of IL-1β and IL-18 (Dib et al., 2015; Mussil et al., 2019; Guo et al., 2020). As mentioned previously, the release of these pro-inflammatory cytokines into the stem cell microenvironment can strongly affect stem cell function and self-renewal. Additionally, mitochondria are a major source of ROS and mitochondrial dysfunction can lead to the release of ROS, which in turn further damages the mitochondria and their mtDNA reinforcing the associated inflammatory response. Interestingly, specific inhibition of mitochondrial ROS was shown to prevent the activation of the NLRP3 inflammasome (Chen et al., 2018).

Furthermore, DSBs of mtDNA upon genotoxic events have been associated with mitochondrial disfunction and consequent mitochondrial RNA (mtRNA) release thereby triggering RIG-1-dependent pathways. Recognition of cytoplasmic mtRNA leads to IFN1 activation and ISG promotion in order to cope with the damaged mtDNA emphasizing the importance of mitochondria-nucleus communication and immunity activation (Tigano et al., 2021). In conclusion, release and recognition of mtDNA upon DSBs can trigger similar immunostimulatory events as the ones observed upon recognition of cytoplasmic self-DNA suggesting that also mtDNA can potentially be implicated in stem cell functional defects and immunity activation. The specific role of mtDNA recognition in inflammation has been recently reviewed by Riley and Tait (2020) (Figure 2).

## Immune Cell Infiltration in the Stem Cell Microenvironment

The immune system works as the main body’s line of defense against pathogens, toxins and tumors while simultaneously being involved in other cellular processes such as tissue development, homeostasis, and repair. The immune response is traditionally classified into innate and adaptive (Vivier and Malissen, 2005). The innate immune cells, such as natural killer cells, mast cells and phagocytic cells, are activated by PAMPs and DAMPs and their non-specific response includes phagocytosis, cell locomotion, killing of pathogens or cells and cytokine production. On the other hand, the adaptive immune response is antigen specific and able to create a long-term immunological memory mediated by cells, like dendritic cells, specialized T cells and B cells (Netea et al., 2020). Interestingly, the innate immune response can be also mediated by stem cells such as HSCs. Indeed, it has been shown that HSCs can create an epigenetic memory in response to genotoxic stress or pathogens and that IFNγ can specifically lead to proliferation and myeloid-biased progenitor differentiation during an innate immune response to infection (Aurora and Olson, 2014; Matatall et al., 2014).

As previously described pathways related to immune cell activation and DNA damage recognition are strongly interconnected and the release of immune modulators upon DNA damage responses can recruit and activate immune cells emulating pathogen infections or tissue injury. An uncontrolled infiltration of immune cells may severely compromise stem cell self-renewal capacity by affecting the local stem cell microenvironment leading to a loss in cellular homeostasis in healthy organs and tissues (Naik et al., 2018). Indeed, it has been observed that infiltration of macrophages can influence skeletal muscle regeneration by affecting the activity and recruitment of fibroadipogenic progenitor cells into the injury site (Low et al., 2017; Dort et al., 2019).

**FIGURE 3:**
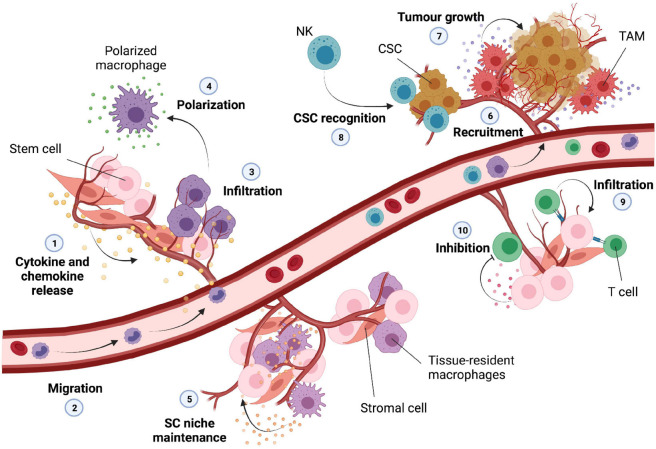
Infiltration of immune cells into the normal stem cell and cancer stem cell niche. DNA damage can trigger the release of various cytokines and chemokines into the microenvironment (1); these can lead to migration and recruitment of macrophages (2) that exit the circulation and infiltrate into the tissue (3). The inflammatory microenvironment is then able to trigger the polarization of these macrophages (4) into M1 or M2 subtypes. Tissue-resident macrophages are normally present into the stem cell microenvironment, and they are important for the maintenance of the stem cell niche (5). The tumor microenvironment can lead to recruitment of various immune cells (6); tumor associated macrophages (TAM) can secrete inflammatory cytokines important for tumor growth and expansion (7); natural killer (NK) cells can potentially recognize and eliminate cancer stem cells (CSC) upon infiltration (8). T-cells can infiltrate into the normal stem cell niche (9) influencing stem cell proliferation and homeostasis; some types of stem cells are able to secrete molecules able to inhibit activation and differentiation of infiltrated T-cells (10).

Macrophages are highly dynamic and essential innate immune cells implicated in tissue homeostasis and regeneration. Their activity is highly controlled by specific signals and their polarization toward a proinflammatory (M1) or anti-inflammatory (M2) phenotype is strongly dependent on the presence of specific cytokines within the environment (Barcellos-Hoff et al., 2005). Pro-inflammatory macrophages are mostly induced my microbial elements as well as TLR ligands and cytokines such as IFNs and TNFα, while anti-inflammatory macrophages can be induced by cytokines like IL-4 and IL-13 (Barcellos-Hoff et al., 2005; Viola et al., 2019). IRFs and NF-κB are the main transcription factors involved in cytokine and chemokine release and chronic activation of these pathways upon DNA damage can thereby influence the recruitment and functionality of unpolarized macrophages (Platanitis and Decker, 2018) (Figure 3, points 1–3).

Other immune regulatory cells, such as MSCs, are able to differentiate into a variety of cell types promoting repair and remodeling of tissues such as bone, cartilage, muscle, adipose and connective tissues (DiMarino et al., 2013; MacDonald and Barrett, 2020). MSCs foster the regeneration of these tissues by controlling immune cell activation, angiogenesis, and extracellular matrix deposition (DiMarino et al., 2013). Recent studies showed that inflammatory cytokines produced by polarized macrophages (Figure 3, point 4) can influence migration and differentiation of human MSCs toward an osteoblastic lineage essential for effective bone tissue regeneration and spinal cord repair (Maldonado-Lasunción et al., 2018; Vallés et al., 2020). Furthermore, it has been shown that MSCs can maintain tissue homeostasis upon injury by inhibiting macrophage activity and T-cell mediated immune responses and thus preserving the activity of corneal epithelial precursor cells (Ko et al., 2020).

Macrophage’s polarization has been suggested to influence not only MSCs but also HSC self-renewal capacity and quiescence status. In fact, macrophages are essential regulators of HSC pool size and mobilization, and the cytokines produced by pro-inflammatory macrophages can directly affect haematopoiesis by acting on the HSC niche and function (McCabe and MacNamara, 2016; Seyfried et al., 2020). It has been described that in physiological conditions tissue resident-macrophages positively contribute to bone marrow homeostasis by promoting HSC niche maintenance and activity. Indeed, these tissue-resident immune cells can produce matrix metalloproteinases in order to degrade the matrix that surrounds HSCs leading to HSC escape into the circulation (Winkler et al., 2010). However, another study showed that resident macrophages are also important for the retention of HSCs in the spleen through the expression of adhesion molecules (Figure 3, point 5). Thus, this demonstrates that elimination of tissue resident macrophages can cause HSC escape into the circulation influencing extramedullary hematopoiesis (Dutta et al., 2015).

The dual proinflammatory and anti-inflammatory feature that macrophages can acquire upon infiltration plays a pivotal role in tissue homeostasis and stem cell function. This seems to be particularly important also for the CSC niche and tumor progression. Macrophage infiltration has been recently associated with many types of tumors and their presence in the tumor environment together with their paracrine signaling have been linked to glioblastoma growth and spread of the CSC phenotype (Shi et al., 2017). Recent works have observed a correlation between the presence of tumor associated macrophages (TAMs) and CSC niche modification and expansion. TAMs are crucial components of the tumor microenvironment, able to exert pro-tumor features through the activation of specific signaling pathways and secretion of a broad range of inflammatory cytokines. For instance, it has been observed that TAMs are able to physically interact with CSCs leading to induction of NF-κB and release of cytokines for the sustainment of the CSC phenotype (Lu et al., 2014). Furthermore, TAMs are also able to promote CSC-like properties through the secretion of higher levels of TGFβ1 compared to other type of macrophages (Fan et al., 2014). Lastly, the presence of TAMs has been positively associated with CSC density in human tumors and the consequent release of pro-inflammatory cytokines has been correlated with an increase of CSC-like cells and invasiveness (Fan et al., 2014) (Figure 3, point 6–7).

Unlike macrophages, natural killer (NK) cells possess abilities to infiltrate and selectively kill CSCs. They are major effectors of innate immunity and therefore able to display a strong cytolytic activity against many tumors or virus-infected cells. Although the role of NK cells in cancer surveillance remains still under debate, recent *in vivo* and *in vitro* studies suggested that NK cells may be able to specifically detect CSCs through the recognition of surface markers leading to a possible decrease in tumor malignity (Tallerico et al., 2017). Furthermore, reduced function of NK cells has been associated with increased risk of developing tumors together with an increased risk in tumor-related mortality (Luna et al., 2017) (Figure 3, point 8).

T cells coordinate multiple adaptive immune responses and are responsible for the recognition of pathogens, antigens and tumors. They originate from bone marrow progenitor cells and upon maturation in the thymus migrate to the periphery of the body in order to exert their patrolling functions. Upon antigen encounter, T cells are able to differentiate into effector cells, a key event for the elimination of pathogens through the production of several cytokines and cytotoxic mediators (Kumar et al., 2018; Goswami and Awasthi, 2020). It has been shown that MSCs can influence T cell proliferation and differentiation *in vitro* through the secretion of a number of soluble factors (Duffy et al., 2011). Galectin-1 in particular is highly expressed by MSCs and able to directly inhibit T cell activation. Indeed, knockdown of this protein *in vitro* has been shown to partially rescue proliferation of both killer and helper T lymphocytes (Gieseke et al., 2010). T cells can differentiate into different subsets of cells; however, dysregulation of this process can lead to immunological deficiencies and autoimmune diseases. For example, it has been observed that NPCs are able to selectively inhibit differentiation of pathogenic T cells through the expression of leukaemia inhibitory factor (LIF) receptors providing potential new insights into multiple sclerosis (Cao et al., 2011). On a separate study, using scRNA sequencing T cells have been shown to infiltrate the adult sub-ventricular zone NSC niche during aging. T cells in old brains secrete IFNγ which promotes a decrease in proliferation of NSCs in both co-culture experiments and *in vivo* (Dulken et al., 2019) (Figure 3, point 9).

Furthermore, MSCs are also able to indirectly modulate T cell activity by affecting maturation and differentiation of antigen presenting cells, such as dendritic cells, important for antigen processing and presentation (Jiang et al., 2005). While neoantigen presentation has been mostly studied in cancer (stem) cells with a focus on T cells and the expression of major histocompatibility complex I (MHC-I) after radiation- or chemotherapy-induced DNA damage in connection with an increased mutational load and subclonal neoantigen generation (McLaughlin et al., 2020), little is known about DNA damage and neoantigen presentation in normal tissue-specific stem cells and their microenvironment (Figure 3, point 10).

## The Dual Role of Cellular Senescence in Stem Cell Function

Cellular senescence is described as an irreversible state of growth arrest triggered by a number of oncogenic events, such as telomere shortening, chromatin perturbation, replication stress, DNA damage and chronic exposure to anti-proliferative cytokines like IFNβ. Unlike quiescence, the senescence state is permanent and cannot be reversed by known physiological stimuli (Campisi and D’Adda Di Fagagna, 2007; Collado et al., 2007). Senescent cells undergo morphological changes accompanying by apoptosis resistance and an altered gene expression pattern that leads to deep metabolic reprogramming and secretion of a wide range of soluble and insoluble factors collectively named senescence-associated secretory phenotype (SASP) (Herranz and Gil, 2018). Furthermore, senescence is associated with persistent DDR activation often correlated with high expression of cell-cycle inhibitors, such as p21 (also termed CDKN1a) and p16 (also termed CDKN2a) (Campisi and D’Adda Di Fagagna, 2007; Faget et al., 2019).

The SASP is a common feature of senescent cells, able to induce paracrine signaling through the secretion of factors including inflammatory cytokines and chemokines. Importantly, it has been observed that there is not a singular SASP phenotype and its composition can change depending on the senescence-inducing triggering factor and cell type (Gonzalez-Meljem et al., 2018). Recent studies have demonstrated that a prolonged DDR activation (Fumagalli et al., 2014) as well as activation of previously mentioned DNA damage-related mechanisms, such as cGAS/STING (Yang et al., 2017), inflammasomes (Yin et al., 2017) and mitochondrial stress pathways (Passos et al., 2007), are able to induce autocrine and paracrine senescence of neighboring cells through the secretion of SASP factors. The promotion of these pathways seems to converge on C/EBPB and NF-κB activation, responsible for the direct regulation of inflammatory cytokine expression and release, in particular IL-8, IL-6 and IL-1α (Herranz and Gil, 2018). IL-1 signaling has been linked to *in vivo* paracrine senescence upon activation of the inflammasome complex (Acosta et al., 2013), while IL-6 and IL-8 are specific interleukins able to act in a paracrine manner to promote senescence development, and their depletion has been shown to prevent senescence entry *in vitro* (Kuilman et al., 2008). Furthermore, STING activation and a sustained IFNβ signaling also cause senescence. Indeed, it has been shown that acute IFNβ stimulation can reversibly arrest cell growth while its chronic stimulation leads to p53-dependent cell cycle arrest and subsequent senescence (Moiseeva et al., 2006).

Although stem cells are able to divide and renew over a long period of time, they are also susceptible to cell cycle arrest and senescence upon exposure to genotoxic stress thereby affecting tissue regeneration and homeostasis (Vitale et al., 2017). A clear example is given by radiation induced-DNA damage, which can promote senescence in different types of stem cells (Chen Z. et al., 2019). In a recent study it has been observed that irradiation induces senescence of bone marrow-derived mesenchymal stem cells (BMSCs), which can be associated with decline in bone formation, a typical side effect of anticancer therapies. Importantly, the secretion of SASP components by senescent BMSCs worsens bone marrow remodeling by inducing osteogenic differentiation dysfunction via paracrine signaling. SASP can therefore be a potential target to ameliorate radiation-induced bone loss (Bai et al., 2020). Although quiescent HSCs are relatively radioresistant, they can be affected by radiation-induced ROS production (Shao et al., 2014; McBride and Schaue, 2020). Indeed, HSCs appear to be quite sensitive to oxidative stress generated upon irradiation or through the activation of proinflammatory pathways (McBride and Schaue, 2020). These events can cause HSCs to undergo premature senescence leading to long-term bone marrow suppression and decrease repopulation capacity (Shao et al., 2014). Cellular senescence has also been identified in NPCs of primary progressive multiple sclerosis (MS)-derived tissue linking DNA damage to remyelination failure and thus offering potential new treatments against MS (Nicaise et al., 2019). In salivary glands irradiation is able to induce accumulation of senescent cells in or near the salivary gland stem and progenitor cell (SGSC) niche both *in vitro* and *in vivo* leading to tissue-specific functional impairment. Selective elimination of senescent cells was shown to improve the self-renewal of SGSCs and to partially rescue salivary secretion activity (Peng et al., 2020). Furthermore, SASP release has been shown to play a pivotal role in salivary gland homeostasis and function upon radiation-induced senescence (Marmary et al., 2016). Sustained expression of IL-6 in particular is known to be essential for both induction of senescence and tissue hypofunction. However, exposure to this cytokine prior irradiation has been shown to enhance DNA repair preventing senescence and salivary gland dysfunction (Marmary et al., 2016). Senescence and SASP promotion can also cause perturbation of the intestinal stem cell niche contributing to potential gastrointestinal disorders, inflammation and carcinogenesis upon heavy ion irradiation and DNA damage (Kumar et al., 2019).

In contrast to normal tissue-specific stem cells, due to its pro-inflammatory features, cellular senescence plays a pivotal role in cancer promotion and stemness. SASP can create an immunosuppressive environment driving tumorigenesis, tumor progression and metastasis (Faget et al., 2019). In multiple myeloma, the release of chemokines, like IP-10 and RANTES, by senescent cells was shown to favor the emergence and maintenance of cancer stem-like cells (Cahu et al., 2012). In CSCs, a gain of stem cell-like features can severely impact tumor progression and aggressiveness. A recent study showed that chemotherapy-induced senescence can lead to a significant upregulation of stem-cell like markers, such as Kit and Sca1, in senescent cells compared to non-senescent cells thereby leading to a much more aggressive tumour phenotype (Milanovic et al., 2018).

Although senescence and SASP are generally associated with aging-related diseases and tumorigenesis, recent studies have also brought to light a positive impact of senescence and SASP in the regeneration and cell reprogramming of some tissues. In acute and chronic muscle injury, cellular plasticity and skeletal muscle reprogramming were shown to be promoted by muscle-damage-induced senescence and SASP release (Chiche et al., 2017). Furthermore, it has been observed that transient exposure to SASP factors promotes cell plasticity, tissue regeneration and stemness while chronic SASP exposure counteracts the regeneration stimuli by inducing cell cycle arrest. Specifically, Ritschka et al. (2017) showed that incubation of primary mouse keratinocytes with conditioned medium from oncogene-induced senescent cells during a short-term period of 2 days led to increased stem cell features and that transplantation of these cells generated more hair follicles compared to untreated cells. However, upon a prolonged exposure to the same SASP both cell-intrinsic and paracrine senescence was observed (Ritschka et al., 2017). Similar findings have been reported by Mosteiro et al. (2018), whose work showed that senescence is important for *in vivo* tissue reprogramming mediated by OCT4, SOX2, KLF4, and MYC (OSKM), four transcription factors used to reprogram somatic cells into iPSCs (Cai et al., 2015; Cevallos et al., 2020). Following this approach, the authors found that paracrine secretion of IL-6 and other soluble factors is essential for the reinforcement of cellular senescence and regeneration upon damage *in vivo* (Mosteiro et al., 2018). This has been recently observed also in fibro-adipogenic progenitor cells highlighting the importance of SASP and senescence in tissue remodeling and plasticity (Saito et al., 2020). Senescence is clearly a powerful mechanism important not only for cell cycle arrest but also for the induction of a state of regenerative inflammation that enhances tissue repair and function upon DNA damage.

## Concluding Remarks

DNA damage profoundly affects the inflammatory microenvironment where stem cells reside, which can have detrimental consequences for their maintenance and long-term function. Indeed, it has been shown that DNA damage-induced immunostimulatory events can lead to tissue-specific stem cell exhaustion leading to degenerative conditions. Conversely, the release of specific cytokines can also positively impact tissue-specific stem cell plasticity and regeneration of damaged tissues in addition to enhance CSC activity leading to tumor progression.

This review provides an overview of the main biological mechanisms linked to changes in the stem cell microenvironment and activation of immune processes upon DNA damage induction. Although recent findings have brought to light new insights into these DNA damage-related inflammatory events, some questions remain unanswered. For instance, it is still not clear how to exploit the production of inflammatory cytokines in order to promote on one side immunostimulatory responses against the tumor and on the other side immunosuppressive responses against aging-related degenerative conditions. Especially since the activation of DNA and RNA sensors might change depending on the specific stimulus and cell type. For example, it has been shown that cytosolic DNA in keratinocytes of psoriatic lesions or exposure of hematopoietic cells to ionizing radiation can both trigger the activation of the AIM2 inflammasome (Dombrowski et al., 2011). However, exposure of keratinocytes to UVB has been shown to induce NLRP3 and cGAS activation with consequent production of a broad spectrum of cytokines including IL-1, IL-6, TNFα, and IFN1 (Hasegawa et al., 2016; Li et al., 2021). These studies exemplified how different stimuli can trigger the activation of different sensors within the same cell type. Whether such observations might also apply to adult stem cells and their self-renewal capacity leading to organ-/tissue-specific outcomes remains to be elucidated.

DNA damage-induced senescence plays a pivotal role in cell cycle arrest and can be used as a barrier against tumor expansion; however, due to the accompanying SASP, it is also responsible for loss of tissue function, aging-related diseases and tumor progression. Therefore, further studies are required to understand how to properly modulate the exposure to SASP factors toward the promotion of a regenerative state and against detrimental effects, such as paracrine senescence of neighboring cells and chronic inflammation. Furthermore, it would be interesting to explore how different types of DNA damage can influence senescence and its SAPS phenotype in different adult stem cells. It has been shown that the SASP associated with radiation-induced DNA damage can differ from SASP induced by other stress factors, such as mitochondrial dysfunction (Wiley et al., 2016; Aratani et al., 2018). Moreover, p53 plays a central role in the DDR and its activity is essential for the prevention of cancer development by promoting cell death and senescence (Mijit et al., 2020). However, it has been shown that in some types of adult stem cells, such as hair follicle bulge stem cells and HSCs, reduced p53 activation upon irradiation mediates resistance to apoptosis or senescence (Sotiropoulou et al., 2010; Insinga et al., 2013) indicating that DDR proteins can actually trigger distinct responses in different types of stem and progenitor cells (Lee et al., 2013).

Further understanding of DNA damage immunomodulatory mechanisms, cell- and stimulus-specific variability might unravel novel strategies to regulate the stem cell microenvironment. Recently developed 3D *in vitro* models, such as organoids and organs-on-a-chip (OOC), represent innovative strategies to address these questions by closely resembling the normal and cancer stem cell microenvironment. Furthermore, organoid co-culture systems and assembloids, organoids generated by incorporating multiple cell types or by fusing organoids of different identities (Vogt, 2021), can be used to study the interaction between immune cells and normal or cancer stem cells upon genotoxic stress. For instance, OOC allow to mimic the combined response to several stimuli and environmental changes by using multiple cell types/stimuli in different chambers (Wu et al., 2020; Almela et al., 2021). These advanced 3D *in vitro* models together with 3D bioprinting techniques, which can reproduce complex tissue structures by using a combination of multicomponent bioinks and cell types (Almela et al., 2021), allow to generate a similar organizational complexity to *in vivo* tissues/organs with the ability to regulate key parameters, such as tissue interactions, concentration gradients and cell patterning (Wu et al., 2020; Almela et al., 2021).

As mentioned in this review, genotoxic stress can affect the stem cell microenvironment leading to stem cell exhaustion, likely through a combination of a decline in cell number and functional capacity, with the emergence of aging-related pathologies (Schumacher et al., 2021). On the other hand, due to their self-renewal properties, CSCs are also affected by DNA damage and the associated inflammatory microenvironment, which can worsen tumor control and treatment efficacy. Understanding the mechanistic links between stem cell properties and microenvironmental changes initiated upon DNA damage will be critical to counteract the functional decline of adult stem cells in aging-related diseases and effectively diminish CSC activity and expansion.

## Author Contributions

DC, RC, and LB wrote the manuscript. DC and LB designed the figures. All authors contributed to the article and approved the submitted version.

## Conflict of Interest

The authors declare that the research was conducted in the absence of any commercial or financial relationships that could be construed as a potential conflict of interest.

## Publisher’s Note

All claims expressed in this article are solely those of the authors and do not necessarily represent those of their affiliated organizations, or those of the publisher, the editors and the reviewers. Any product that may be evaluated in this article, or claim that may be made by its manufacturer, is not guaranteed or endorsed by the publisher.

## References

[B1] Ablasser A. Chen Z. J. (2019). CGAS in action: expanding roles in immunity and inflammation. *Science* 363:eaat8657. 10.1126/science.aat8657 30846571

[B2] Acosta J. C. Banito A. Wuestefeld T. Georgilis A. Janich P. Morton J. P. (2013). A complex secretory program orchestrated by the inflammasome controls paracrine senescence. *Nat. Cell Biol.* 15 978–990. 10.1038/ncb2784 23770676 PMC3732483

[B3] Adamiak M. Abdel-Latif A. Bujko K. Thapa A. Anusz K. Tracz M. (2020). Nlrp3 inflammasome signaling regulates the homing and engraftment of hematopoietic stem cells (HSPCs) by enhancing incorporation of CXCR4 receptor into membrane lipid rafts. *Stem Cell Rev. Rep.* 165 954–967. 10.1007/S12015-020-10005-W 32661868 PMC7456406

[B4] Ahn J.-S. Seo Y. Oh S.-J. Yang J. W. Shin Y. Y. Lee B.-C. (2020). The activation of NLRP3 inflammasome potentiates the immunomodulatory abilities of mesenchymal stem cells in a murine colitis model. *BMB Rep.* 53 329–334. 10.5483/BMBREP.2020.53.6.065 32475381 PMC7330809

[B5] Almela T. Tayebi L. Moharamzadeh K. (2021). 3D bioprinting for in vitro models of oral cancer: toward development and validation. *Bioprinting* 22:e00132. 10.1016/J.BPRINT.2021.E00132 34368488 PMC8341396

[B6] Almine J. F. O’Hare C. A. J. Dunphy G. Haga I. R. Naik R. J. Atrih A. (2017). IFI16 and cGAS cooperate in the activation of STING during DNA sensing in human keratinocytes. *Nat. Commun.* 8:14392. 10.1038/ncomms14392 28194029 PMC5316833

[B7] Aratani S. Tagawa M. Nagasaka S. Sakai Y. Shimizu A. Tsuruoka S. (2018). Radiation-induced premature cellular senescence involved in glomerular diseases in rats. *Sci. Rep.* 8:16812. 10.1038/s41598-018-34893-8 30429495 PMC6235850

[B8] Aurora A. B. Olson E. N. (2014). Immune modulation of stem cells and regeneration. *Cell Stem Cell* 15 14–25. 10.1016/j.stem.2014.06.009 24996166 PMC4131296

[B9] Bai J. Wang Y. Wang J. Zhai J. He F. Zhu G. (2020). Irradiation-induced senescence of bone marrow mesenchymal stem cells aggravates osteogenic differentiation dysfunction via paracrine signaling. *Am. J. Physiol. Cell Physiol.* 318 C1005–C1017. 10.1152/ajpcell.00520.2019 32233952

[B10] Barazzuol L. Ju L. Jeggo P. A. (2017). A coordinated DNA damage response promotes adult quiescent neural stem cell activation. *PLoS Biol.* 15:e2001264. 10.1371/JOURNAL.PBIO.2001264 28489848 PMC5424956

[B11] Barcellos-Hoff M. H. Park C. Wright E. G. (2005). Radiation and the microenvironment – tumorigenesis and therapy. *Nat. Rev. Cancer* 5 867–875. 10.1038/nrc1735 16327765

[B12] Behrens A. Van Deursen J. M. Rudolph K. L. Schumacher B. (2014). Impact of genomic damage and ageing on stem cell function. *Nat. Cell Biol.* 16 201–207. 10.1038/ncb2928 24576896 PMC4214082

[B13] Bergsbaken T. Fink S. L. Cookson B. T. (2009). Pyroptosis: host cell death and inflammation. *Nat. Rev. Microbiol.* 7 99–109. 10.1038/nrmicro2070 19148178 PMC2910423

[B14] Biechonski S. Yassin M. Milyavsky M. (2017). DNA-damage response in hematopoietic stem cells: an evolutionary trade-off between blood regeneration and leukemia suppression. *Carcinogenesis* 38 367–377. 10.1093/CARCIN/BGX002 28334174

[B15] Blanpain C. Simons B. D. (2013). Unravelling stem cell dynamics by lineage tracing. *Nat. Rev. Mol. Cell Biol.* 14 489–502. 10.1038/nrm3625 23860235

[B16] Burger K. Schlackow M. Potts M. Hester S. Mohammed S. Gullerova M. (2017). Nuclear phosphorylated Dicer processes double-stranded RNA in response to DNA damage. *J. Cell Biol.* 216 2373–2389. 10.1083/JCB.201612131 28642363 PMC5551710

[B17] Cahu J. Bustany S. Sola B. (2012). Senescence-associated secretory phenotype favors the emergence of cancer stem-like cells. *Cell Death Dis.* 3:446. 10.1038/cddis.2012.183 23254289 PMC3542619

[B18] Cai Y. Dai X. Zhang Q. Dai Z. (2015). Gene expression of OCT4, SOX2, KLF4 and MYC (OSKM) induced pluripotent stem cells: identification for potential mechanisms. *Diagn. Pathol.* 10:35. 10.1186/S13000-015-0263-7 25907774 PMC4414430

[B19] Campisi J. D’Adda Di Fagagna F. (2007). Cellular senescence: when bad things happen to good cells. *Nat. Rev. Mol. Cell Biol.* 8 729–740. 10.1038/nrm2233 17667954

[B20] Cao W. Yang Y. Wang Z. Liu A. Fang L. Wu F. (2011). Leukemia inhibitory factor inhibits T helper 17 cell differentiation and confers treatment effects of neural progenitor cell therapy in autoimmune disease. *Immunity* 35 273–284. 10.1016/j.immuni.2011.06.011 21835648

[B21] Castellani C. A. Longchamps R. J. Sun J. Guallar E. Arking D. E. (2020). Thinking outside the nucleus: mitochondrial DNA copy number in health and disease. *Mitochondrion* 53 214–223. 10.1016/j.mito.2020.06.004 32544465 PMC7375936

[B22] Cevallos R. R. Edwards Y. J. K. Parant J. M. Yoder B. K. Hu K. (2020). Human transcription factors responsive to initial reprogramming predominantly undergo legitimate reprogramming during fibroblast conversion to iPSCs. *Sci. Rep.* 10:19710. 10.1038/s41598-020-76705-y 33184372 PMC7661723

[B23] Chaudhuri J. Bains Y. Guha S. Kahn A. Hall D. Bose N. (2018). The role of advanced glycation end products in aging and metabolic diseases: bridging association and causality. *Cell Metab.* 28:337. 10.1016/J.CMET.2018.08.014 30184484 PMC6355252

[B24] Chen M. Reed R. R. Lane A. P. (2019). Chronic inflammation directs an olfactory stem cell functional switch from neuroregeneration to immune defense. *Cell Stem Cell* 25 501–513.e5. 10.1016/j.stem.2019.08.011 31523027 PMC6778045

[B25] Chen Y. Zhou Z. Min W. (2018). Mitochondria, oxidative stress and innate immunity. *Front. Physiol.* 9:1487. 10.3389/FPHYS.2018.01487 30405440 PMC6200916

[B26] Chen Z. Cao K. Xia Y. Li Y. Hou Y. Wang L. (2019). Cellular senescence in ionizing radiation (Review). *Oncol. Rep.* 42 883–894. 10.3892/or.2019.7209 31233195

[B27] Chiche A. Le Roux I. von Joest M. Sakai H. Aguín S. B. Cazin C. (2017). Injury-induced senescence enables in vivo reprogramming in skeletal muscle. *Cell Stem Cell* 20 407–414.e4. 10.1016/j.stem.2016.11.020 28017795

[B28] Choi M. E. Price D. R. Ryter S. W. Choi A. M. K. (2019). Necroptosis: a crucial pathogenic mediator of human disease. *JCI Insight* 4:e128834. 10.1172/jci.insight.128834 31391333 PMC6693822

[B29] Clapes T. Polyzou A. Prater P. Morales-Hernández A. Galvao Ferrarini M. Kehrer N. (2021). Chemotherapy-induced transposable elements activate MDA5 to enhance haematopoietic regeneration. *Nat. Cell Biol.* 23 704–717. 10.1038/s41556-021-00707-9 34253898 PMC8492473

[B30] Collado M. Blasco M. A. Serrano M. (2007). Cellular senescence in cancer and aging. *Cell* 130 223–233. 10.1016/j.cell.2007.07.003 17662938

[B31] Coppes R. P. Dubrovska A. (2017). Targeting stem cells in radiation oncology. *Clin. Oncol.* 29 329–334. 10.1016/j.clon.2017.03.005 28363465

[B32] Crane G. M. Jeffery E. Morrison S. J. (2017). Adult haematopoietic stem cell niches. *Nat. Rev. Immunol.* 17 573–590. 10.1038/nri.2017.53 28604734

[B33] Crasta K. Ganem N. J. Dagher R. Lantermann A. B. Ivanova E. V. Pan Y. (2012). DNA breaks and chromosome pulverization from errors in mitosis. *Nature* 482 53–58. 10.1038/nature10802 22258507 PMC3271137

[B34] Crop M. J. Baan C. C. Korevaar S. S. IJzermans J. N. M. Pescatori M. Stubbs A. P. (2010). Inflammatory conditions affect gene expression and function of human adipose tissue-derived mesenchymal stem cells. *Clin. Exp. Immunol.* 162 474–486. 10.1111/j.1365-2249.2010.04256.x 20846162 PMC3026550

[B35] Detmer S. A. Chan D. C. (2007). Functions and dysfunctions of mitochondrial dynamics. *Nat. Rev. Mol. Cell Biol.* 8 870–879. 10.1038/nrm2275 17928812

[B36] Devos M. Tanghe G. Gilbert B. Dierick E. Verheirstraeten M. Nemegeer J. (2020). Sensing of endogenous nucleic acids by ZBP1 induces keratinocyte necroptosis and skin inflammation. *J. Exp. Med.* 217:e20191913. 10.1084/jem.20191913 32315377 PMC7336309

[B37] Dib B. Lin H. Maidana D. E. Tian B. Miller J. B. Bouzika P. (2015). Mitochondrial DNA has a pro-inflammatory role in AMD. *Biochim. Biophys. Acta Mol. Cell Res.* 1853 2897–2906. 10.1016/j.bbamcr.2015.08.012 26305120 PMC5330253

[B38] DiMarino A. M. Caplan A. I. Bonfield T. L. (2013). Mesenchymal stem cells in tissue repair. *Front. Immunol.* 4:201. 10.3389/fimmu.2013.00201 24027567 PMC3761350

[B39] Dinarello C. A. (2018). Overview of the IL-1 family in innate inflammation and acquired immunity. *Immunol. Rev.* 281 8–27. 10.1111/imr.12621 29247995 PMC5756628

[B40] Doherty M. R. Cheon H. Junk D. J. Vinayak S. Varadan V. Telli M. L. (2017). Interferon-beta represses cancer stem cell properties in triple-negative breast cancer. *Proc. Natl. Acad. Sci. U.S.A.* 114 13792–13797. 10.1073/pnas.1713728114 29229854 PMC5748193

[B41] Dombrowski Y. Peric M. Koglin S. Kammerbauer C. Göß C. Anz D. (2011). Cytosolic DNA triggers inflammasome activation in keratinocytes in psoriatic lesions. *Sci. Transl. Med.* 3:82ra38. 10.1126/SCITRANSLMED.3002001 21562230 PMC3235683

[B42] Dort J. Fabre P. Molina T. Dumont N. A. (2019). Macrophages are key regulators of stem cells during skeletal muscle regeneration and diseases. *Stem Cells Int.* 2019 1–20. 10.1155/2019/4761427 31396285 PMC6664695

[B43] Duffy M. M. Ritter T. Ceredig R. Griffin M. D. (2011). Mesenchymal stem cell effects on T-cell effector pathways. *Stem Cell Res. Ther.* 2:34. 10.1186/scrt75 21861858 PMC3219065

[B44] Dulken B. W. Buckley M. T. Navarro Negredo P. Saligrama N. Cayrol R. Leeman D. S. (2019). Single-cell analysis reveals T cell infiltration in old neurogenic niches. *Nature* 571 205–210. 10.1038/s41586-019-1362-5 31270459 PMC7111535

[B45] Dunphy G. Flannery S. M. Almine J. F. Connolly D. J. Paulus C. Jønsson K. L. (2018). Non-canonical activation of the DNA sensing adaptor STING by ATM and IFI16 Mediates NF-κB signaling after nuclear DNA damage. *Mol. Cell* 71 745–760.e5. 10.1016/j.molcel.2018.07.034 30193098 PMC6127031

[B46] Dupont S. Morsut L. Aragona M. Enzo E. Giulitti S. Cordenonsi M. (2011). Role of YAP/TAZ in mechanotransduction. *Nature* 474 179–184. 10.1038/nature10137 21654799

[B47] Dutta P. Hoyer F. F. Grigoryeva L. S. Sager H. B. Leuschner F. Courties G. (2015). Macrophages retain hematopoietic stem cells in the spleen via VCAM-1. *J. Exp. Med.* 212 497–512. 10.1084/jem.20141642 25800955 PMC4387283

[B48] Faget D. V. Ren Q. Stewart S. A. (2019). Unmasking senescence: context-dependent effects of SASP in cancer. *Nat. Rev. Cancer* 19 439–453. 10.1038/s41568-019-0156-2 31235879

[B49] Fan Q. M. Jing Y. Y. Yu G. F. Kou X. R. Ye F. Gao L. (2014). Tumor-associated macrophages promote cancer stem cell-like properties via transforming growth factor-beta1-induced epithelial-mesenchymal transition in hepatocellular carcinoma. *Cancer Lett.* 352 160–168. 10.1016/j.canlet.2014.05.008 24892648

[B50] Fang E. Scheibye-Knudsen M. Brace L. Kassahun H. SenGupta T. Nilsen H. (2014). Defective mitophagy in XPA via PARP-1 hyperactivation and NAD(+)/SIRT1 reduction. *Cell* 157 882–896. 10.1016/J.CELL.2014.03.026 24813611 PMC4625837

[B51] Fernandes-Alnemri T. Yu J. W. Datta P. Wu J. Alnemri E. S. (2009). AIM2 activates the inflammasome and cell death in response to cytoplasmic DNA. *Nature* 458, 509–513. 10.1038/nature07710 19158676 PMC2862225

[B52] Flach J. Bakker S. T. Mohrin M. Conroy P. C. Pietras E. M. Reynaud D. (2014). Replication stress is a potent driver of functional decline in ageing haematopoietic stem cells. *Nature* 512 198–202. 10.1038/nature13619 25079315 PMC4456040

[B53] Foell D. Wittkowski H. Roth J. (2007). Mechanisms of disease: a “DAMP” view of inflammatory arthritis. *Nat. Clin. Pract. Rheumatol.* 3 382–390. 10.1038/ncprheum0531 17599072

[B54] Fumagalli M. Rossiello F. Mondello C. D’Adda Di Fagagna F. (2014). Stable cellular senescence is associated with persistent DDR activation. *PLoS One* 9:110969. 10.1371/journal.pone.0110969 25340529 PMC4207795

[B55] Gao Y. Yu X. F. Chen T. (2021). Human endogenous retroviruses in cancer: expression, regulation and function (Review). *Oncol. Lett.* 21:121. 10.3892/ol.2020.12382 33552242 PMC7798031

[B56] Gasteiger G. D’osualdo A. Schubert D. A. Weber A. Bruscia E. M. Hartl D. (2017). Cellular innate immunity: an old game with new players. *J. Innate Immun.* 9 111–125. 10.1159/000453397 28006777 PMC6738785

[B57] Ghosh R. Roy S. Franco S. (2018). PARP1 depletion induces RIG-I-dependent signaling in human cancer cells. *PLoS One* 13:e0194611. 10.1371/journal.pone.0194611 29590171 PMC5874037

[B58] Gieseke F. Böhringer J. Bussolari R. Dominici M. Handgretinger R. Müller I. (2010). Human multipotent mesenchymal stromal cells use galectin-1 to inhibit immune effector cells. *Blood* 116 3770–3779. 10.1182/blood-2010-02-270777 20644118

[B59] Gong T. Liu L. Jiang W. Zhou R. (2019). DAMP-sensing receptors in sterile inflammation and inflammatory diseases. *Nat. Rev. Immunol.* 20 95–112. 10.1038/s41577-019-0215-7 31558839

[B60] Gonzalez-Meljem J. M. Apps J. R. Fraser H. C. Martinez-Barbera J. P. (2018). Paracrine roles of cellular senescence in promoting tumourigenesis. *Br. J. Cancer* 118 1283–1288. 10.1038/s41416-018-0066-1 29670296 PMC5959857

[B61] Goswami R. Awasthi A. (2020). Editorial: T cell differentiation and function in tissue inflammation. *Front. Immunol.* 11:289. 10.3389/fimmu.2020.00289 32153592 PMC7047510

[B62] Guo H. Callaway J. B. Ting J. P. Y. (2015). Inflammasomes: mechanism of action, role in disease, and therapeutics. *Nat. Med.* 21 677–687. 10.1038/nm.3893 26121197 PMC4519035

[B63] Guo Y. Gu R. Gan D. Hu F. Li G. Xu G. (2020). Mitochondrial DNA drives noncanonical inflammation activation via cGAS–STING signaling pathway in retinal microvascular endothelial cells. *Cell Commun. Signal.* 18 1–12. 10.1186/s12964-020-00637-3 33115500 PMC7592595

[B64] Gurung C. Fendereski M. Sapkota K. Guo J. Huang F. Guo Y. L. (2021). Dicer represses the interferon response and the double-stranded RNA-activated protein kinase pathway in mouse embryonic stem cells. *J. Biol. Chem.* 296:100264. 10.1016/J.JBC.2021.100264 33837743 PMC7948645

[B65] Hasegawa T. Nakashima M. Suzuki Y. (2016). Nuclear DNA damage-triggered NLRP3 inflammasome activation promotes UVB-induced inflammatory responses in human keratinocytes. *Biochem. Biophys. Res. Commun.* 477 329–335. 10.1016/J.BBRC.2016.06.106 27343554

[B66] Hatch E. M. Fischer A. H. Deerinck T. J. Hetzer M. W. (2013). Catastrophic nuclear envelope collapse in cancer cell micronuclei. *Cell* 154 47–60. 10.1016/J.CELL.2013.06.007 23827674 PMC3749778

[B67] Hayden M. S. West A. P. Ghosh S. (2006). NF-κB and the immune response. *Oncogene* 25 6758–6780. 10.1038/sj.onc.1209943 17072327

[B68] He Y. Hara H. Núñez G. (2016). Mechanism and regulation of NLRP3 inflammasome activation. *Trends Biochem. Sci.* 41, 1012–1021. 10.1016/j.tibs.2016.09.002 27669650 PMC5123939

[B69] Herranz N. Gil J. (2018). Mechanisms and functions of cellular senescence. *J. Clin. Invest.* 128 1238–1246. 10.1172/JCI95148 29608137 PMC5873888

[B70] Hormaechea-Agulla D. Le D. T. King K. Y. (2020). Common sources of inflammation and their impact on hematopoietic stem cell biology. *Curr. Stem Cell Rep.* 6 96–107. 10.1007/s40778-020-00177-z 32837857 PMC7429415

[B71] Hornung V. Ablasser A. Charrel-Dennis M. Bauernfeind F. Horvath G. Caffrey D. R. (2009). AIM2 recognizes cytosolic dsDNA and forms a caspase-1-activating inflammasome with ASC. *Nature* 458, 514–518. 10.1038/nature07725 19158675 PMC2726264

[B72] Hu B. Jin C. Li H. B. Tong J. Ouyang X. Cetinbas N. M. (2016). The DNA-sensing AIM2 inflammasome controls radiation-induced cell death and tissue injury. *Science* 354 765–768. 10.1126/science.aaf7532 27846608 PMC5640175

[B73] Huang C.-F. Chen L. Li Y.-C. Wu L. Yu G.-T. Zhang W.-F. (2017). NLRP3 inflammasome activation promotes inflammation-induced carcinogenesis in head and neck squamous cell carcinoma. *J. Exp. Clin. Cancer Res.* 36 1–13. 10.1186/S13046-017-0589-Y 28865486 PMC5581464

[B74] Iliou M. S. da Silva-Diz V. Carmona F. J. Ramalho-Carvalho J. Heyn H. Villanueva A. (2014). Impaired DICER1 function promotes stemness and metastasis in colon cancer. *Oncogene* 33:4003. 10.1038/ONC.2013.398 24096488 PMC4114136

[B75] Inomata K. Aoto T. Binh N. T. Okamoto N. Tanimura S. Wakayama T. (2009). Genotoxic stress abrogates renewal of melanocyte stem cells by triggering their differentiation. *Cell* 137 1088–1099. 10.1016/J.CELL.2009.03.037 19524511

[B76] Inoue M. Shinohara M. L. (2013). NLRP3 inflammasome and MS/EAE. *Autoimmune Dis.* 2013:859145. 10.1155/2013/859145 23365725 PMC3556409

[B77] Insinga A. Cicalese A. Faretta M. Gallo B. Albano L. Ronzoni S. (2013). DNA damage in stem cells activates p21, inhibits p53, and induces symmetric self-renewing divisions. *Proc. Natl. Acad. Sci. U.S.A.* 110 3931–3936. 10.1073/PNAS.1213394110 23417300 PMC3593901

[B78] Ishikawa H. Barber G. N. (2008). STING is an endoplasmic reticulum adaptor that facilitates innate immune signalling. *Nature* 455 674–678. 10.1038/nature07317 18724357 PMC2804933

[B79] Ishikawa H. Ma Z. Barber G. N. (2009). STING regulates intracellular DNA-mediated, type i interferon-dependent innate immunity. *Nature* 461 788–792. 10.1038/nature08476 19776740 PMC4664154

[B80] Jackson S. P. Bartek J. (2009). The DNA-damage response in human biology and disease. *Nature* 461 1071–1078. 10.1038/nature08467 19847258 PMC2906700

[B81] Jahandideh B. Derakhshani M. Abbaszadeh H. Akbar Movassaghpour A. Mehdizadeh A. Talebi M. (2020). The pro-inflammatory cytokines effects on mobilization, self-renewal and differentiation of hematopoietic stem cells. *Hum. Immunol.* 81 206–217. 10.1016/j.humimm.2020.01.004 32139091

[B82] Jefferies C. A. (2019). Regulating IRFs in IFN driven disease. *Front. Immunol.* 10:325. 10.3389/fimmu.2019.00325 30984161 PMC6449421

[B83] Jeggo P. A. Pearl L. H. Carr A. M. (2016). DNA repair, genome stability and cancer: a historical perspective. *Nat. Rev. Cancer* 16 35–42. 10.1038/nrc.2015.4 26667849

[B84] Jeong Y. J. Oh H. K. Park S. H. Bong J. G. (2018). Association between inflammation and cancer stem cell phenotype in breast cancer. *Oncol. Lett.* 15 2380–2386. 10.3892/ol.2017.7607 29434947 PMC5777276

[B85] Jiang X. X. Zhang Y. Liu B. Zhang S. X. Wu Y. Yu X. D. (2005). Human mesenchymal stem cells inhibit differentiation and function of monocyte-derived dendritic cells. *Blood* 105 4120–4126. 10.1182/blood-2004-02-0586 15692068

[B86] Jones D. L. Wagers A. J. (2008). No place like home: anatomy and function of the stem cell niche. *Nat. Rev. Mol. Cell Biol.* 9 11–21. 10.1038/nrm2319 18097443

[B87] Jounai N. Kobiyama K. Takeshita F. Ishii K. J. (2013). Recognition of damage-associated molecular patterns related to nucleic acids during inflammation and vaccination. *Front. Cell. Infect. Microbiol.* 2:168. 10.3389/fcimb.2012.00168 23316484 PMC3539075

[B88] Kawase-Koga Y. Low R. Otaegi G. Pollock A. Deng H. Eisenhaber F. (2010). RNAase-III enzyme Dicer maintains signaling pathways for differentiation and survival in mouse cortical neural stem cells. *J. Cell Sci.* 123 586–594. 10.1242/JCS.059659 20103535 PMC2818196

[B89] Keith B. Simon M. C. (2007). Hypoxia-inducible factors, stem cells, and cancer. *Cell* 129 465–472. 10.1016/j.cell.2007.04.019 17482542 PMC3150586

[B90] Kenyon J. Gerson S. L. (2007). The role of DNA damage repair in aging of adult stem cells. *Nucleic Acids Res.* 35 7557–7565. 10.1093/NAR/GKM1064 18160407 PMC2190724

[B91] Kingham E. Oreffo R. O. C. (2013). Embryonic and induced pluripotent stem cells: understanding, creating, and exploiting the nano-niche for regenerative medicine. *ACS Nano* 7:1867. 10.1021/NN3037094 23414366 PMC3610401

[B92] Ko J. H. Kim J. Jeong H. J. Lee H. J. Youn J. Correspondence O. (2020). Mesenchymal stem and stromal cells harness macrophage-derived amphiregulin to maintain tissue homeostasis. *Cell Rep.* 30 3806–3820.e6. 10.1016/j.celrep.2020.02.062 32187551

[B93] Koltsova A. S. Pendina A. A. Efimova O. A. Chiryaeva O. G. Kuznetzova T. V. Baranov V. S. (2019). On the complexity of mechanisms and consequences of chromothripsis: an update. *Front. Genet.* 10:393. 10.3389/FGENE.2019.00393 31114609 PMC6503150

[B94] Kuehbacher A. Urbich C. Zeiher A. M. Dimmeler S. (2007). Role of Dicer and drosha for endothelial microRNA expression and angiogenesis. *Circ. Res.* 101 59–68. 10.1161/CIRCRESAHA.107.153916 17540974

[B95] Kuilman T. Michaloglou C. Vredeveld L. C. W. Douma S. van Doorn R. Desmet C. J. (2008). Oncogene-induced senescence relayed by an interleukin-dependent inflammatory network. *Cell* 133 1019–1031. 10.1016/j.cell.2008.03.039 18555778

[B96] Kumar B. V. Connors T. J. Farber D. L. (2018). Human T cell development, localization, and function throughout life. *Immunity* 48 202–213. 10.1016/j.immuni.2018.01.007 29466753 PMC5826622

[B97] Kumar S. Suman S. Fornace A. J. Datta K. (2019). Intestinal stem cells acquire premature senescence and senescence associated secretory phenotype concurrent with persistent DNA damage after heavy ion radiation in mice. *Aging* 11 4145–4158. 10.18632/aging.102043 31239406 PMC6629005

[B98] Kuriakose T. Kanneganti T. D. (2018). ZBP1: innate sensor regulating cell death and inflammation. *Trends Immunol.* 39 123–134. 10.1016/j.it.2017.11.002 29236673 PMC5863909

[B99] Kwon M. Leibowitz M. L. Lee J.-H. (2020). Small but mighty: the causes and consequences of micronucleus rupture. *Exp. Mol. Med.* 52 1777–1786. 10.1038/s12276-020-00529-z 33230251 PMC8080619

[B100] Lane S. W. Williams D. A. Watt F. M. (2014). Modulating the stem cell niche for tissue regeneration. *Nat. Biotechnol.* 32 795–803. 10.1038/nbt.2978 25093887 PMC4422171

[B101] Lee A. J. Ashkar A. A. (2018). The dual nature of type I and type II interferons. *Front. Immunol.* 9:2061. 10.3389/fimmu.2018.02061 30254639 PMC6141705

[B102] Lee A. K. Pan D. Bao X. Hu M. Li F. Li C. Y. (2020). Endogenous retrovirus activation as a key mechanism of anti-tumor immune response in radiotherapy. *Radiat. Res.* 193 305–317. 10.1667/RADE-20-00013 32074012 PMC7359417

[B103] Lee C.-L. Blum J. M. Kirsch D. G. (2013). Role of p53 in regulating tissue response to radiation by mechanisms independent of apoptosis. *Transl. Cancer Res.* 2 412–421. 10.21037/1638 24466508 PMC3898670

[B104] Li C. Liu W. Wang F. Hayashi T. Mizuno K. Hattori S. (2021). DNA damage-triggered activation of cGAS-STING pathway induces apoptosis in human keratinocyte HaCaT cells. *Mol. Immunol.* 131 180–190. 10.1016/J.MOLIMM.2020.12.037 33423764

[B105] Li D. Wu R. Guo W. Xie L. Qiao Z. Chen S. (2019). STING-mediated IFI16 degradation negatively controls type I interferon production. *Cell Rep.* 29 1249–1260.e4. 10.1016/j.celrep.2019.09.069 31665637

[B106] Li L. Li L. Zhang Z. Jiang Z. (2015). Hypoxia promotes bone marrow-derived mesenchymal stem cell proliferation through apelin/APJ/autophagy pathway. *Acta Biochim. Biophys. Sin.* 47 362–367. 10.1093/abbs/gmv014 25736405

[B107] Li T. Chen Z. J. (2018). The cGAS-cGAMP-STI NG pathway connects DNA damage to inflammation, senescence, and cancer. *J. Exp. Med.* 215 1287–1299. 10.1084/jem.20180139 29622565 PMC5940270

[B108] Li W. Lu L. Lu J. Wang X. Yang C. Jin J. (2020). cGAS-STING-mediated DNA sensing maintains CD8+ T cell stemness and promotes antitumor T cell therapy. *Sci. Transl. Med.* 12:eaay9013. 10.1126/scitranslmed.aay9013 32581136

[B109] Liao W. Du C. Wang J. (2020). The cGAS-STING pathway in hematopoiesis and its physiopathological significance. *Front. Immunol.* 11:573915. 10.3389/fimmu.2020.573915 33329537 PMC7734179

[B110] Lin J.-Y. Kuo R.-L. Huang H.-I. (2019). Activation of type I interferon antiviral response in human neural stem cells. *Stem Cell Res. Ther.* 10 1–17. 10.1186/S13287-019-1521-5 31843025 PMC6916114

[B111] Liu T. Zhang L. Joo D. Sun S. C. (2017). NF-κB signaling in inflammation. *Signal Transduct. Target. Ther.* 2:17023. 10.1038/sigtrans.2017.23 29158945 PMC5661633

[B112] Liu X. Zhang Z. Ruan J. Pan Y. Magupalli V. G. Wu H. (2016). Inflammasome-activated gasdermin D causes pyroptosis by forming membrane pores. *Nature* 535 153–158. 10.1038/nature18629 27383986 PMC5539988

[B113] Low M. Eisner C. Rossi F. (2017). “Fibro/adipogenic progenitors (FAPs): isolation by FACS and culture,” in *Methods in Molecular Biology*, eds Perdiguero E. Cornelison D. (New York, NY: Humana Press Inc.), 179–189. 10.1007/978-1-4939-6771-1_9 28247350

[B114] Lu H. Clauser K. R. Tam W. L. Fröse J. Ye X. Eaton E. N. (2014). A breast cancer stem cell niche supported by juxtacrine signalling from monocytes and macrophages. *Nat. Cell Biol.* 16 1105–1117. 10.1038/ncb3041 25266422 PMC4296514

[B115] Luna J. I. Grossenbacher S. K. Murphy W. J. Canter R. J. (2017). Targeting cancer stem cells with natural killer cell immunotherapy. *Expert Opin. Biol. Ther.* 17 313–324. 10.1080/14712598.2017.1271874 27960589 PMC5311007

[B116] MacDonald E. S. Barrett J. G. (2020). The potential of mesenchymal stem cells to treat systemic inflammation in horses. *Front. Vet. Sci.* 6:507. 10.3389/fvets.2019.00507 32039250 PMC6985200

[B117] MacKenzie K. J. Carroll P. Martin C. A. Murina O. Fluteau A. Simpson D. J. (2017). CGAS surveillance of micronuclei links genome instability to innate immunity. *Nature* 548 461–465. 10.1038/nature23449 28738408 PMC5870830

[B118] Madabhushi R. Pan L. Tsai L. H. (2014). DNA damage and its links to neurodegeneration. *Neuron* 83 266–282. 10.1016/j.neuron.2014.06.034 25033177 PMC5564444

[B119] Maekawa H. Inoue T. Ouchi H. Jao T. Inoue R. Nishi H. (2019). Mitochondrial damage causes inflammation *via* cGAS-STING signaling in acute kidney injury. *Cell Rep.* 29, 1261–1273. 10.1016/j.celrep.2019.09.050 31665638

[B120] Maldonado-Lasunción I. Verhaagen J. Oudega M. (2018). Mesenchymal stem cell-macrophage choreography supporting spinal cord repair. *Neurotherapeutics* 15 578–587. 10.1007/s13311-018-0629-0 29728851 PMC6095786

[B121] Man S. M. Zhu Q. Zhu L. Liu Z. Karki R. Malik A. (2015). Critical role for the DNA sensor AIM2 in stem cell proliferation and cancer. *Cell* 162 45–58. 10.1016/J.CELL.2015.06.001 26095253 PMC4491002

[B122] Mandal P. Blanpain C. Rossi D. (2011). DNA damage response in adult stem cells: pathways and consequences. *Nat. Rev. Mol. Cell Biol.* 12 198–202. 10.1038/NRM3060 21304553

[B123] Marmary Y. Adar R. Gaska S. Wygoda A. Maly A. Cohen J. (2016). Radiation-induced loss of salivary gland function is driven by cellular senescence and prevented by IL6 modulation. *Cancer Res.* 76 1170–1180. 10.1158/0008-5472.CAN-15-1671 26759233

[B124] Martin-Hijano L. Sainz B. (2020). The interactions between cancer stem cells and the innate interferon signaling pathway. *Front. Immunol.* 11:526. 10.3389/fimmu.2020.00526 32296435 PMC7136464

[B125] Matatall K. A. Shen C. C. Challen G. A. King K. Y. (2014). Type II interferon promotes differentiation of myeloid-biased hematopoietic stem cells. *Stem Cells* 32 3023–3030. 10.1002/stem.1799 25078851 PMC4198460

[B126] Matsumura H. Mohri Y. Binh N. T. Morinaga H. Fukuda M. Ito M. (2016). Hair follicle aging is driven by transepidermal elimination of stem cells via COL17A1 proteolysis. *Science* 351:aad4395. 10.1126/SCIENCE.AAD4395 26912707

[B127] McBride W. H. Schaue D. (2020). Radiation-induced tissue damage and response. *J. Pathol.* 250 647–655. 10.1002/path.5389 31990369 PMC7216989

[B128] McCabe A. MacNamara K. C. (2016). Macrophages: key regulators of steady-state and demand-adapted hematopoiesis. *Exp. Hematol.* 44 213–222. 10.1016/j.exphem.2016.01.003 26806720 PMC4852701

[B129] McLaughlin M. Patin E. C. Pedersen M. Wilkins A. Dillon M. T. Melcher A. A. (2020). Inflammatory microenvironment remodelling by tumour cells after radiotherapy. *Nat. Rev. Cancer* 20 203–217. 10.1038/s41568-020-0246-1 32161398

[B130] Mendelson A. Frenette P. S. (2014). Hematopoietic stem cell niche maintenance during homeostasis and regeneration. *Nat. Med.* 20 833–846. 10.1038/nm.3647 25100529 PMC4459580

[B131] Mijit M. Caracciolo V. Melillo A. Amicarelli F. Giordano A. (2020). Role of p53 in the regulation of cellular senescence. *Biomolecules* 10:420. 10.3390/BIOM10030420 32182711 PMC7175209

[B132] Milanovic M. Fan D. N. Y. Belenki D. Däbritz J. H. M. Zhao Z. Yu Y. (2018). Senescence-associated reprogramming promotes cancer stemness. *Nature* 553 96–100. 10.1038/nature25167 29258294

[B133] Moiseeva O. Mallette F. A. Mukhopadhyay U. K. Moores A. Ferbeyre G. (2006). DNA damage signaling and p53-dependent senescence after prolonged β-interferon stimulation. *Mol. Biol. Cell* 17 1583–1592. 10.1091/mbc.E05-09-0858 16436515 PMC1415317

[B134] Mosteiro L. Pantoja C. de Martino A. Serrano M. (2018). Senescence promotes in vivo reprogramming through p16 INK4a and IL-6. *Aging Cell* 17:e12711. 10.1111/acel.12711 29280266 PMC5847859

[B135] Much C. Auchynnikava T. Pavlinic D. Buness A. Rappsilber J. Benes V. (2016). Endogenous mouse Dicer is an exclusively cytoplasmic protein. *PLoS Genet.* 12:e1006095. 10.1371/JOURNAL.PGEN.1006095 27254021 PMC4890738

[B136] Mussil B. Suspène R. Caval V. Durandy A. Wain-Hobson S. Vartanian J. P. (2019). Genotoxic stress increases cytoplasmic mitochondrial DNA editing by human APOBEC3 mutator enzymes at a single cell level. *Sci. Rep.* 9:3109. 10.1038/s41598-019-39245-8 30816165 PMC6395610

[B137] Naik S. Larsen S. B. Cowley C. J. Fuchs E. (2018). Two to Tango: dialog between immunity and stem cells in health and disease. *Cell* 175 908–920. 10.1016/j.cell.2018.08.071 30388451 PMC6294328

[B138] Naik S. Larsen S. B. Gomez N. C. Alaverdyan K. Sendoel A. Yuan S. (2017). Inflammatory memory sensitizes skin epithelial stem cells to tissue damage. *Nature* 550 475–480. 10.1038/nature24271 29045388 PMC5808576

[B139] Nakad R. Schumacher B. (2016). DNA damage response and immune defense: links and mechanisms. *Front. Genet.* 7:147. 10.3389/fgene.2016.00147 27555866 PMC4977279

[B140] Netea M. G. Domínguez-Andrés J. Barreiro L. B. Chavakis T. Divangahi M. Fuchs E. (2020). Defining trained immunity and its role in health and disease. *Nat. Rev. Immunol.* 20 375–388. 10.1038/s41577-020-0285-6 32132681 PMC7186935

[B141] Nicaise A. M. Wagstaff L. J. Willis C. M. Paisie C. Chandok H. Robson P. (2019). Cellular senescence in progenitor cells contributes to diminished remyelination potential in progressive multiple sclerosis. *Proc. Natl. Acad. Sci. U.S.A.* 116 9030–9039. 10.1073/pnas.1818348116 30910981 PMC6500153

[B142] Onomoto K. Onoguchi K. Yoneyama M. (2021). Regulation of RIG-I-like receptor-mediated signaling: interaction between host and viral factors. *Cell. Mol. Immunol.* 18 539–555. 10.1038/s41423-020-00602-7 33462384 PMC7812568

[B143] Orvain C. Lin Y.-L. Jean-Louis F. Hocini H. Hersant B. Bennasser Y. (2020). Hair follicle stem cell replication stress drives IFI16/STING-dependent inflammation in hidradenitis suppurativa. *J. Clin. Invest.* 130:3777. 10.1172/JCI131180 32240121 PMC7324185

[B144] Paludan S. R. Bowie A. G. (2013). Immune sensing of DNA. *Immunity* 38 870–880. 10.1016/j.immuni.2013.05.004 23706668 PMC3683625

[B145] Park E. J. Shimaoka M. Kiyono H. (2017). MicroRNA-mediated dynamic control of mucosal immunity. *Int. Immunol.* 29 157–163. 10.1093/INTIMM/DXX019 28383678

[B146] Passos J. F. Saretzki G. Ahmed S. Nelson G. Richter T. Peters H. (2007). Mitochondrial dysfunction accounts for the stochastic heterogeneity in telomere-dependent senescence. *PLoS Biol.* 5:e110. 10.1371/journal.pbio.0050110 17472436 PMC1858712

[B147] Pazhanisamy S. K. (2009). Stem cells, DNA damage, ageing and cancer. *Hematol. Oncol. Stem Cell Ther.* 2 375–384. 10.1016/S1658-3876(09)50005-2 20139050

[B148] Peng X. Wu Y. Brouwer U. van Vliet T. Wang B. Demaria M. (2020). Cellular senescence contributes to radiation-induced hyposalivation by affecting the stem/progenitor cell niche. *Cell Death Dis.* 11:854. 10.1038/s41419-020-03074-9 33056980 PMC7566836

[B149] Pennings S. Liu K. J. Qian H. (2018). The stem cell niche: Interactions between stem cells and their environment. *Stem Cells Int.* 2018:4879379. 10.1155/2018/4879379 30405721 PMC6204189

[B150] Piccaluga P. P. Agostinelli C. Fuligni F. Righi S. Tripodo C. Re M. C. (2015). IFI16 expression is related to selected transcription factors during B-cell differentiation. *J. Immunol. Res.* 10.1155/2015/747645 26185770 PMC4491573

[B151] Ping Z. Chen S. Hermans S. J. F. Kenswil K. J. G. Feyen J. van Dijk C. (2019). Activation of NF-κB driven inflammatory programs in mesenchymal elements attenuates hematopoiesis in low-risk myelodysplastic syndromes. *Leukemia* 33 536–541. 10.1038/s41375-018-0267-x 30315231 PMC6365382

[B152] Pinho S. Frenette P. S. (2019). Haematopoietic stem cell activity and interactions with the niche. *Nat. Rev. Mol. Cell Biol.* 20 303–320. 10.1038/s41580-019-0103-9 30745579 PMC6483843

[B153] Platanitis E. Decker T. (2018). Regulatory networks involving STATs, IRFs, and NFκB in inflammation. *Front. Immunol.* 9:2542. 10.3389/fimmu.2018.02542 30483250 PMC6242948

[B154] Poirier E. Z. Buck M. D. Chakravarty P. Carvalho J. Frederico B. Cardoso A. (2021). An isoform of Dicer protects mammalian stem cells against multiple RNA viruses. *Science* 373 231–236. 10.1126/SCIENCE.ABG2264 34244417 PMC7611482

[B155] Pronk C. J. H. Veiby O. P. Bryder D. Jacobsen S. E. W. (2011). Tumor necrosis factor restricts hematopoietic stem cell activity in mice: involvement of two distinct receptors. *J. Exp. Med.* 208 1563–1570. 10.1084/JEM.20110752 21768269 PMC3149225

[B156] Qian P. He X. C. Paulson A. Li Z. Tao F. Perry J. M. (2016). The Dlk1-Gtl2 locus preserves LT-HSC function by inhibiting the PI3K-mTOR pathway to restrict mitochondrial metabolism. *Cell Stem Cell* 18 214–228. 10.1016/J.STEM.2015.11.001 26627594 PMC5545934

[B157] Rehwinkel J. Gack M. U. (2020). RIG-I-like receptors: their regulation and roles in RNA sensing. *Nat. Rev. Immunol.* 20 537–551. 10.1038/s41577-020-0288-3 32203325 PMC7094958

[B158] Riley J. S. Tait S. W. (2020). Mitochondrial DNA in inflammation and immunity. *EMBO Rep.* 21:e49799. 10.15252/embr.201949799 32202065 PMC7132203

[B159] Ritschka B. Storer M. Mas A. Heinzmann F. Ortells M. C. Morton J. P. (2017). The senescence-associated secretory phenotype induces cellular plasticity and tissue regeneration. *Genes Dev.* 31 172–183. 10.1101/gad.290635.116 28143833 PMC5322731

[B160] Sagulenko V. Thygesen S. Sester D. Idris A. Cridland J. A. Vajjhala P. R. (2013). AIM2 and NLRP3 inflammasomes activate both apoptotic and pyroptotic death pathways *via* ASC. *Cell Death Differ.* 20, 1149–1160. 10.1038/cdd.2013.37 23645208 PMC3741496

[B161] Saito Y. Chikenji T. S. Matsumura T. Nakano M. Fujimiya M. (2020). Exercise enhances skeletal muscle regeneration by promoting senescence in fibro-adipogenic progenitors. *Nat. Commun.* 11:889. 10.1038/s41467-020-14734-x 32060352 PMC7021787

[B162] Sato T. Onai N. Yoshihara H. Arai F. Suda T. Ohteki T. (2009). Interferon regulatory factor-2 protects quiescent hematopoietic stem cells from type i interferon-dependent exhaustion. *Nat. Med.* 15 696–700. 10.1038/nm.1973 19483695

[B163] Scadden D. T. (2006). The stem-cell niche as an entity of action. *Nature* 441 1075–1079. 10.1038/nature04957 16810242

[B164] Schroder K. Tschopp J. (2010). The inflammasomes. *Cell* 140 821–832. 10.1016/j.cell.2010.01.040 20303873

[B165] Schumacher B. Pothof J. Vijg J. Hoeijmakers J. H. J. (2021). The central role of DNA damage in the ageing process. *Nature* 592 695–703. 10.1038/s41586-021-03307-7 33911272 PMC9844150

[B166] Scully R. Panday A. Elango R. Willis N. A. (2019). DNA double-strand break repair-pathway choice in somatic mammalian cells. *Nat. Rev. Mol. Cell Biol.* 20 698–714. 10.1038/s41580-019-0152-0 31263220 PMC7315405

[B167] Seldin L. Macara I. G. (2020). DNA damage promotes epithelial hyperplasia and fate mis-specification via fibroblast inflammasome activation. *Dev. Cell* 55 558–573.e6. 10.1016/j.devcel.2020.09.021 33058780 PMC7725994

[B168] Sémont A. François S. Mouiseddine M. François A. Saché A. Frick J. (2006). Mesenchymal stem cells increase self-renewal of small intestinal epithelium and accelerate structural recovery after radiation injury. *Adv. Exp. Med. Biol.* 585 19–30. 10.1007/978-0-387-34133-0_2 17120774

[B169] Seyfried A. N. Maloney J. M. MacNamara K. C. (2020). Macrophages orchestrate hematopoietic programs and regulate HSC function during inflammatory stress. *Front. Immunol.* 11:1499. 10.3389/fimmu.2020.01499 32849512 PMC7396643

[B170] Shang D. Yang P. Liu Y. Song J. Zhang F. Tian Y. (2011). Interferon-α induces G1 cell-cycle arrest in renal cell carcinoma cells via activation of Jak-Stat signaling. *Cancer Invest.* 29 347–352. 10.3109/07357907.2011.568566 21599510

[B171] Shao L. Feng W. Li H. Gardner D. Luo Y. Wang Y. (2014). Total body irradiation causes long-term mouse BM injury via induction of HSC premature senescence in an Ink4a-and Arf-independent manner. *Blood* 123 3105–3115. 10.1182/blood-2013-07 24622326 PMC4023419

[B172] Sharma M. Rajendrarao S. Shahani N. Ramírez-Jarquín U. N. Subramaniam S. (2020). Cyclic GMP-AMP synthase promotes the inflammatory and autophagy responses in Huntington disease. *Proc. Natl. Acad. Sci. U.S.A.* 117 15989–15999. 10.1073/PNAS.2002144117 32581130 PMC7354937

[B173] Sharma M. de Alba E. (2021). Structure, activation and regulation of NLRP3 and AIM2 inflammasomes. *Int. J. Mol. Sci.* 22:872. 10.3390/ijms22020872 33467177 PMC7830601

[B174] Shevde N. (2012). Stem cells: flexible friends. *Nature* 483 S22–S26. 10.1038/483s22a 22378125

[B175] Shi Y. Ping Y. F. Zhou W. He Z. C. Chen C. Bian B. S. J. (2017). Tumour-associated macrophages secrete pleiotrophin to promote PTPRZ1 signalling in glioblastoma stem cells for tumour growth. *Nat. Commun.* 8:15080. 10.1038/ncomms15080 28569747 PMC5461490

[B176] Shin T.-H. Kim H.-S. Choi S. W. Kang K.-S. (2017). Mesenchymal stem cell therapy for inflammatory skin diseases: clinical potential and mode of action. *Int. J. Mol. Sci.* 18:244. 10.3390/IJMS18020244 28125063 PMC5343781

[B177] Silva-Gomes S. Decout A. Nigou J. (2014). “Pathogen-associated molecular patterns (PAMPs),” in *Encyclopedia of Inflammatory Diseases*, ed. Parnham M. (Basel: Birkhäuser), 1–16. 10.1007/978-3-0348-0620-6_35-1

[B178] Singh G. Pachouri U. C. Khaidem D. C. Kundu A. Chopra C. Singh P. (2015). Mitochondrial DNA damage and diseases. *F1000Res.* 4:176. 10.12688/f1000research.6665.1 27508052 PMC4962287

[B179] Sotiropoulou P. A. Candi A. Mascré G. De Clercq S. Youssef K. K. Lapouge G. (2010). Bcl-2 and accelerated DNA repair mediates resistance of hair follicle bulge stem cells to DNA-damage-induced cell death. *Nat. Cell Biol.* 12 572–582. 10.1038/ncb2059 20473297

[B180] Strowig T. Henao-Mejia J. Elinav E. Flavell R. (2012). Inflammasomes in health and disease. *Nature* 481 278–286. 10.1038/nature10759 22258606

[B181] Sullivan C. B. Porter R. M. Evans C. H. Ritter T. Shaw G. Barry F. (2014). TNFα and IL-1β influence the differentiation and migration of murine MSCs independently of the NF-κB pathway. *Stem Cell Res. Ther.* 5:104. 10.1186/scrt492 25163844 PMC4177434

[B182] Takaoka A. Wang Z. Choi M. K. Yanai H. Negishi H. Ban T. (2007). DAI (DLM-1/ZBP1) is a cytosolic DNA sensor and an activator of innate immune response. *Nature* 448 501–505. 10.1038/nature06013 17618271

[B183] Tallerico R. Conti L. Lanzardo S. Sottile R. Garofalo C. Wagner A. K. (2017). NK cells control breast cancer and related cancer stem cell hematological spread. *Oncoimmunology* 6:e1284718. 10.1080/2162402X.2017.1284718 28405511 PMC5384375

[B184] Tigano M. Vargas D. C. Tremblay-Belzile S. Fu Y. Sfeir A. (2021). Nuclear sensing of breaks in mitochondrial DNA enhances immune surveillance. *Nature* 591 477–481. 10.1038/s41586-021-03269-w 33627873

[B185] Tiwari V. Wilson III D. M. (2019). DNA damage and associated DNA repair defects in disease and premature aging. *Am. J. Hum. Genet.* 105 237–257. 10.1016/j.ajhg.2019.06.005 31374202 PMC6693886

[B186] Tulotta C. Lefley D. V. Freeman K. Gregory W. M. Hanby A. M. Heath P. R. (2019). Endogenous production of IL1B by breast cancer cells drives metastasis and colonization of the bone microenvironment. *Clin. Cancer Res.* 25 2769–2782. 10.1158/1078-0432.CCR-18-2202 30670488

[B187] Vallés G. Bensiamar F. Maestro-Paramio L. García-Rey E. Vilaboa N. Saldaña L. (2020). Influence of inflammatory conditions provided by macrophages on osteogenic ability of mesenchymal stem cells. *Stem Cell Res. Ther.* 11:57. 10.1186/s13287-020-1578-1 32054534 PMC7020593

[B188] Van Gorp H. Lamkanfi M. (2019). The emerging roles of inflammasome−dependent cytokines in cancer development. *EMBO Rep.* 20:e47575. 10.15252/embr.201847575 31101676 PMC6549028

[B189] Vescovi A. L. Galli R. Reynolds B. A. (2006). Brain tumour stem cells. *Nat. Rev. Cancer* 6 425–436. 10.1038/nrc1889 16723989

[B190] Viola A. Munari F. Sánchez-Rodríguez R. Scolaro T. Castegna A. (2019). The metabolic signature of macrophage responses. *Front. Immunol.* 10:1462. 10.3389/fimmu.2019.01462 31333642 PMC6618143

[B191] Vishlaghi N. Lisse T. S. (2020). Dicer- and bulge stem cell-dependent microRNAs during induced anagen hair follicle development. *Front. Cell Dev. Biol.* 8:338. 10.3389/FCELL.2020.00338 32478074 PMC7240072

[B192] Vitale I. Manic G. De Maria R. Kroemer G. Galluzzi L. (2017). DNA damage in stem cells. *Mol. Cell* 66 306–319. 10.1016/J.MOLCEL.2017.04.006 28475867

[B193] Vivier E. Malissen B. (2005). Innate and adaptive immunity: specificities and signaling hierarchies revisited. *Nat. Immunol.* 6 17–21. 10.1038/ni1153 15611777 PMC7097365

[B194] Vogt N. (2021). Assembloids. *Nat. Methods* 18:27. 10.1038/s41592-020-01026-x 33408387

[B195] Vono R. Garcia E. J. Spinetti G. Madeddu P. (2018). Oxidative stress in mesenchymal stem cell senescence: regulation by coding and noncoding RNAs. *Antioxid. Redox Signal.* 29 864–879. 10.1089/ARS.2017.7294 28762752 PMC6080119

[B196] Voog J. Jones D. L. (2010). Stem cells and the niche: a dynamic duo. *Cell Stem Cell* 6 103–115. 10.1016/j.stem.2010.01.011 20144784 PMC3012646

[B197] Wagers A. J. Weissman I. L. (2004). Plasticity of adult stem cells. *Cell* 116 639–648. 10.1016/S0092-8674(04)00208-9 15006347

[B198] Wang R. Li H. Wu J. Cai Z.-Y. Li B. Ni H. (2020). Gut stem cell necroptosis by genome instability triggers bowel inflammation. *Nature* 580 386–390. 10.1038/s41586-020-2127-x 32296174

[B199] Wei J. Wang H. Wang H. Wang B. Meng L. Xin Y. (2019). The role of NLRP3 inflammasome activation in radiation damage. *Biomed. Pharmacother.* 118:109217. 10.1016/j.biopha.2019.109217 31302424

[B200] White E. Schlackow M. Kamieniarz-Gdula K. Proudfoot N. J. Gullerova M. (2014). Human nuclear Dicer restricts the deleterious accumulation of endogenous double-stranded RNA. *Nat. Struct. Mol. Biol.* 21 552–559. 10.1038/nsmb.2827 24814348 PMC4129937

[B201] Widera D. Kaus A. Kaltschmidt C. Kaltschmidt B. (2008). Neural stem cells, inflammation and NF-κB: basic principle of maintenance and repair or origin of brain tumours? Neuroscience review series. *J. Cell. Mol. Med.* 12 459–470. 10.1111/j.1582-4934.2007.00208.x 18182066 PMC3822535

[B202] Wiley C. Velarde M. Lecot P. Liu S. Sarnoski E. Freund A. (2016). Mitochondrial dysfunction induces senescence with a distinct secretory phenotype. *Cell Metab.* 23 303–314. 10.1016/J.CMET.2015.11.011 26686024 PMC4749409

[B203] Winkler I. G. Sims N. A. Pettit A. R. Barbier V. Nowlan B. Helwani F. (2010). Bone marrow macrophages maintain hematopoietic stem cell (HSC) niches and their depletion mobilizes HSCs. *Blood* 116 4815–4828. 10.1182/blood-2009-11-253534 20713966

[B204] Wu Q. Liu J. Wang X. Feng L. Wu J. Zhu X. (2020). Organ-on-a-chip: recent breakthroughs and future prospects. *Biomed. Eng. OnLine* 19 1–19. 10.1186/S12938-020-0752-0 32050989 PMC7017614

[B205] Xia P. Wang S. Gao P. Gao G. Fan Z. (2016). DNA sensor cGAS-mediated immune recognition. *Protein Cell* 7, 777–791. 10.1007/s13238-016-0320-3 27696330 PMC5084157

[B206] Yang H. Wang H. Ren U. Chen Q. Chena Z. J. (2017). CGAS is essential for cellular senescence. *Proc. Natl. Acad. Sci. U.S.A.* 114 E4612–E4620. 10.1073/pnas.1705499114 28533362 PMC5468617

[B207] Yang K. Wang J. Wu M. Li M. Wang Y. Huang X. (2015). Mesenchymal stem cells detect and defend against gammaherpesvirus infection via the cGAS-STING pathway. *Sci. Rep.* 5:7820. 10.1038/srep07820 25592282 PMC4296288

[B208] Yang K. Wang J. Xiang A. P. Zhan X. Wang Y. Wu M. (2013). Functional RIG-I-like receptors control the survival of mesenchymal stem cells. *Cell Death Dis.* 4:e967. 10.1038/CDDIS.2013.504 24336087 PMC3877571

[B209] Yin Y. Zhou Z. Liu W. Chang Q. Sun G. Dai Y. (2017). Vascular endothelial cells senescence is associated with NOD-like receptor family pyrin domain-containing 3 (NLRP3) inflammasome activation via reactive oxygen species (ROS)/thioredoxin-interacting protein (TXNIP) pathway. *Int. J. Biochem. Cell Biol.* 84 22–34. 10.1016/j.biocel.2017.01.001 28064010

[B210] Yu Q. Katlinskaya Y. V. Carbone C. J. Zhao B. Katlinski K. V. Zheng H. (2015). DNA-damage-induced type I interferon promotes senescence and inhibits stem cell function. *Cell Rep.* 11 785–797. 10.1016/j.celrep.2015.03.069 25921537 PMC4426031

[B211] Yum S. Li M. Fang Y. Chen Z. J. (2021). TBK1 recruitment to STING activates both IRF3 and NF-κB that mediate immune defense against tumors and viral infections. *Proc. Natl. Acad. Sci. U.S.A.* 118:2100225118. 10.1073/pnas.2100225118 33785602 PMC8040795

[B212] Zhang D. Liu C. Li H. Jiao J. (2020). Deficiency of STING signaling in embryonic cerebral cortex leads to neurogenic abnormalities and autistic-like behaviors. *Adv. Sci.* 7:2002117. 10.1002/advs.202002117 33304758 PMC7710002

[B213] Zhang H. Menzies K. J. Auwerx J. (2018). The role of mitochondria in stem cell fate and aging. *Development* 145:dev143420. 10.1242/DEV.143420 29654217 PMC5964648

[B214] Zhang S. Yang X. Wang L. Zhang C. (2018). Interplay between inflammatory tumor microenvironment and cancer stem cells (Review). *Oncol. Lett.* 16 679–686. 10.3892/ol.2018.8716 29963133 PMC6019921

[B215] Zhao C. Zhao W. (2020). NLRP3 inflammasome—A key player in antiviral responses. *Front. Immunol.* 11:211. 10.1016/j.jhep.2016.12.018 32133002 PMC7040071

[B216] Zhao X. Xie L. Wang Z. Wang J. Xu H. Han X. (2020). ZBP1 (DAI/DLM-1) promotes osteogenic differentiation while inhibiting adipogenic differentiation in mesenchymal stem cells through a positive feedback loop of Wnt/β-catenin signaling. *Bone Res.* 8:12. 10.1038/s41413-020-0085-4 PMC7058036 32195010

[B217] Zheng Z. Jia S. Shao C. Shi Y. (2020). Irradiation induces cancer lung metastasis through activation of the cGAS–STING–CCL5 pathway in mesenchymal stromal cells. *Cell Death Dis.* 11:326. 10.1038/s41419-020-2546-5 32382015 PMC7206094

